# Exosomes Derived from HIV-1 Infected DCs Mediate Viral trans-Infection via Fibronectin and Galectin-3

**DOI:** 10.1038/s41598-017-14817-8

**Published:** 2017-11-01

**Authors:** Rutuja Kulkarni, Anil Prasad

**Affiliations:** Division of Experimental Medicine, Beth Israel Deaconess Medical Center, Harvard Medical School, Boston, USA

## Abstract

Exosomes are membrane enclosed nano-sized vesicles actively released into the extracellular milieu that can harbor genomic, proteomic and lipid cargos. Functionally, they are shown to regulate cell-cell communication and transmission of pathogens. Though studies have implicated a role for exosomes in HIV-1 pathogenesis, their mechanisms are not well defined. Here, we characterized exosomes derived from uninfected or HIV-1 infected T-cells and DCs. We demonstrate substantial differences in morphological, molecular and biogenesis machinery between exosomes derived from these two immune cell types. In addition, exosomes derived from HIV-1 infected DCs were 4 fold more infective than either cell free HIV-1 or exosomes derived from T-cells. Molecular analysis of exosomes detected the presence of fibronectin and galectin-3 in those derived from DCs, whereas T-cell exosomes lacked these molecules. Addition of anti-fibronectin antibody and β-lactose, a galectin-3 antagonist, significantly blocked DC exosome-mediated HIV-1 infection of T-cells. We also observed increased gene expression of the pro-inflammatory cytokines IFN-γ, TNF-α, IL-1β and RANTES and activation of p38/Stat pathways in T-cells exposed to exosomes derived from HIV-1 infected DCs. Our study provides insight into the role of exosomes in HIV pathogenesis and suggests they can be a target in development of novel therapeutic strategies against viral infection.

## Introduction

While there have been notable advances in combatting the AIDS epidemic, HIV-1 infection remains a global health problem due to lack of an effective vaccine and frequent treatment failure^1,2^. This highlights the need to better understand the mechanisms involved in host-pathogen interaction, particularly viral immune evasion and cell-to-cell transmission. Escape of virus from immune detection may occur by altering the host cellular trafficking machinery, specifically inducing formation of cytoskeletal structures such as nanotubes and infectious synapses^3–8^. Recently, another mechanism involving ‘Trojan exosomes’ has been implicated in viral spread and immune activation^9–13^.

Exosomes are extracellular nanovesicles (30–200 nm in diameter) formed by the inward budding of the endosomal compartments, resulting in multivesicular bodies (MVBs) that are released upon fusion with the plasma membrane^14–17^. Actively secreted by various cell types, exosomes have been successfully isolated from various body fluids such as urine, saliva, bile, breast milk or blood and from cell culture medium^13,18–23^. They can carry proteins, lipids and nucleic acids; however their cargo mainly depends on physiological conditions and their origin^24–26^. Exosomes may act as regulators of both innate and acquired immunity by stimulating cytokine production, inflammatory responses and antigen presentation^18,27–29^. In addition, exosomes have been shown to play roles in viral pathogenesis by altering host defense mechanisms and facilitating dissemination of the microbes^11,13,28,30–32^. Analysis of exosomes derived from HIV-1 infected cells has revealed the presence of various viral components, including the viral genome^9^. Those derived from HIV-1 infected macrophages and dendritic cells (DCs) can transfer infection to uninfected cells and induce robust viral replication^13,31^. When derived from CCR5 or CXCR4 positive cells, exosomes appear to transfer these HIV-1 co-receptors to CCR5 or CXCR4 negative cells and may make them susceptible to infection^33,34^. Furthermore, exosome-mediated transfer of HIV-1 nef to host cells can alter the intracellular trafficking machinery, enhance HIV-1 replication and release, and increase formation of MVBs^35–37^. Further, exposure to exosomes containing HIV-1 nef and ADAM17 transformed resting CD4+ T cells, making them permissive for HIV-1 infection, and may trigger apoptosis^38^. Under some conditions, exosomes may prevent viral infection by activating immune cells and inducing anti-viral adaptive immune responses^11,18,39^. In this context, exosomes can transfer intrinsic resistance factors such as APOBEC3G from cell to cell and enhance resistance to HIV-1 infection^40^. Exosomes isolated from human semen and breast milk have shown to inhibit HIV-1 replication and cell-to-cell transmission of virus^41,42^.

Here, we characterize exosomes derived from HIV-1 infected and uninfected T cells and DCs and demonstrate that those derived from DCs can transfer HIV-1 to T cells and facilitate robust replication through fibronectin and galectin-3 mediated cellular fusion. Further, we show that such exosomes can induce production of pro-inflammatory cytokines. These novel observations provide insights into how virus may modulate host immune responses via exosomes to the benefit of the pathogen.

## Results

### Comparison of exosomes derived from T cells and DCs

**Figure 1:**
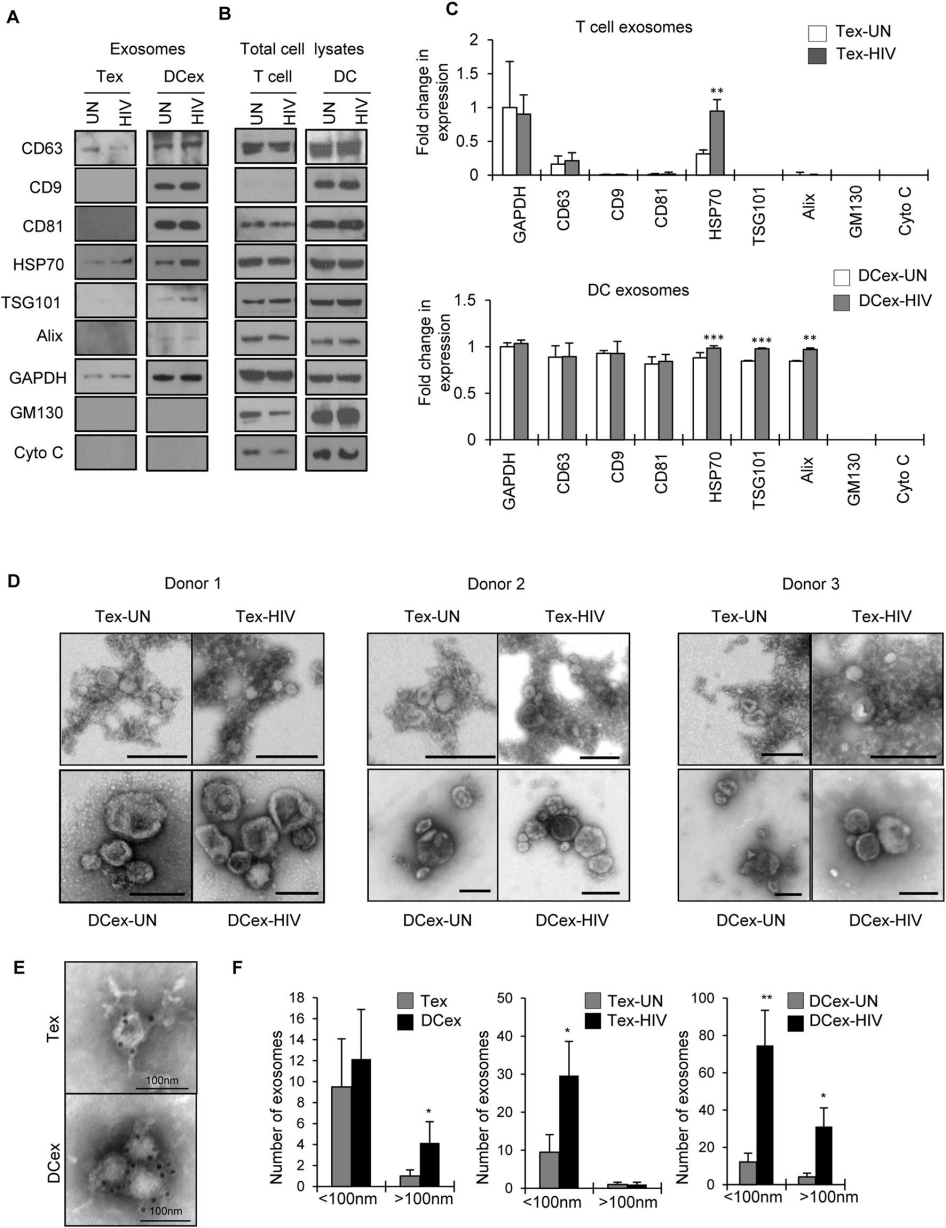
Characterization of exosomes. (**A**) Exosomes from uninfected and HIV-1 infected T cells (Tex-UN and Tex-HIV) or DCs (DCex-UN and DCex-HIV) were lysed and 10 µg of lysates were analyzed for indicated exosome markers by Western blotting. GAPDH served as a loading control. (**B**) Representative Western blot images of indicated markers in total cell lysates of uninfected or HIV-1 infected T cells and DCs. Full-length blots are presented in Supplementary Figure S3. (**C**) Quantitative analysis of Western blot images of (**A**); the band intensity of each lane was determined by Image J software and pixel density was calculated. Fold change was determined by considering band intensity of GAPDH in untreated cells as 1. (**p ≤ 0.01, ***p ≤ 0.001, p-values for Tex HSP70 – 0.0036, DCex HSP70 – 0.00004; TSG101 – 0.00005; Alix – 0.0087). (**D**) Electron microscopic images of negatively stained Tex-UN, Tex-HIV, DCex-UN and DCex-HIV from 3 different donor preparations. Scale bar represents 200 nm. (**E**) Electron microscopic images showing CD63 Immuno-labeling in exosomes (Scale bar - 100 nm). (**F**) Bar diagrams showing the size distribution of exosomes quantitated by electron microscopic images from uninfected T cells (Tex) and DCs (DCex) (left panel), Tex-UN and Tex-HIV (middle panel), and DCex-UN and DCex-HIV (right panel). The number of exosomes in each size range (nm) is the mean from 10 different fields of electron microscope images (*p ≤ 0.05, **p ≤ 0.01, p-values for DCex > 100 nm – 0.044807, Tex-HIV < 100 nm – 0.02558, DCex-HIV < 100 nm – 0.00505, DCex-HIV > 100 nm – 0.01016).

We first analyzed exosomes isolated from uninfected or HIV-1 infected T cells and DCs by examining the exosome markers CD63, CD9, CD81 and HSP70^28^. Western blot analysis revealed increased expression of these markers in exosomes derived from DCs compared to those from T cells. However, we did not observe significant differences in the expression pattern of these markers between exosomes derived from HIV-1 infected DCs compared to those derived from uninfected cells except HSP70, with markedly increased expression in exosomes derived from virus infected DCs (Fig. 1A and C). Surprisingly, when we analyzed exosomes isolated from T cells, we did not observe expression of CD81 and CD9 markers, but found weak expression of CD63 and HSP70 (Fig. 1A). Analysis of molecules involved in multivesicular endosome biogenesis revealed the expression of TSG101 and Alix in exosomes derived from DCs; expression of TSG101 markedly increased in exosomes derived from HIV-1 infected DCs compared to those from uninfected DCs. Exosomes derived from T cells lacked expression of TSG101 while a low level of Alix expression was observed. GAPDH was used as a loading control, however exosomes derived from T cells exhibited decreased expression of GAPDH compared to those of DCs, despite loading equal amounts of protein. We also analyzed expression of these molecules in total cell lysates and observed presence of these molecules in the cell lysates of both cell types (Fig. 1B). Previous studies have demonstrated the absence of GM130 (Golgi apparatus marker) and cytochrome C (mitochondrial marker) in the exosomes and hence these molecules may be considered as negative markers for exosomes^43,44^. We analyzed expression of these markers in our exosome preparations and observed that both T cell and DC exosomes lacked these markers (Fig. 1A). However, total cell lysates of both cell types showed the expression of these molecules (Fig. 1B). Further, electron microscopic (EM) analysis of exosome preparations derived from both T cells and DCs revealed that T cells produced uniformly sized vesicles of 20–100 nm diameters with low electron density in the center. However, exosomes derived from DCs were pleomorphic with sizes varying from 20–300 nm. We did not observe differences in morphology between exosomes derived from HIV-1 infected and uninfected cells. We also confirmed these results in exosomes derived from three different donors (Fig. 1D). Furthermore, to confirm the CD63 positivity in these exosome preparations we performed EM analysis with immunogold staining using anti-CD63 antibodies. The EM images confirmed CD63 expression in exosomes derived from both T cells and DCs (Fig. 1E). On quantification of exosomes based on EM images, we observed a significant increase in number of exosomes released from DCs compared to T cells (Fig. 1F, left panel). Further, we observed a significant increase in exosome release from infected T cells and DCs compared to uninfected cells (Fig. 1F, middle and right panels).

### CD63 +ve exosomes derived from HIV-1 infected DCs mediated enhanced HIV-1 trans- infection

**Figure 2:**
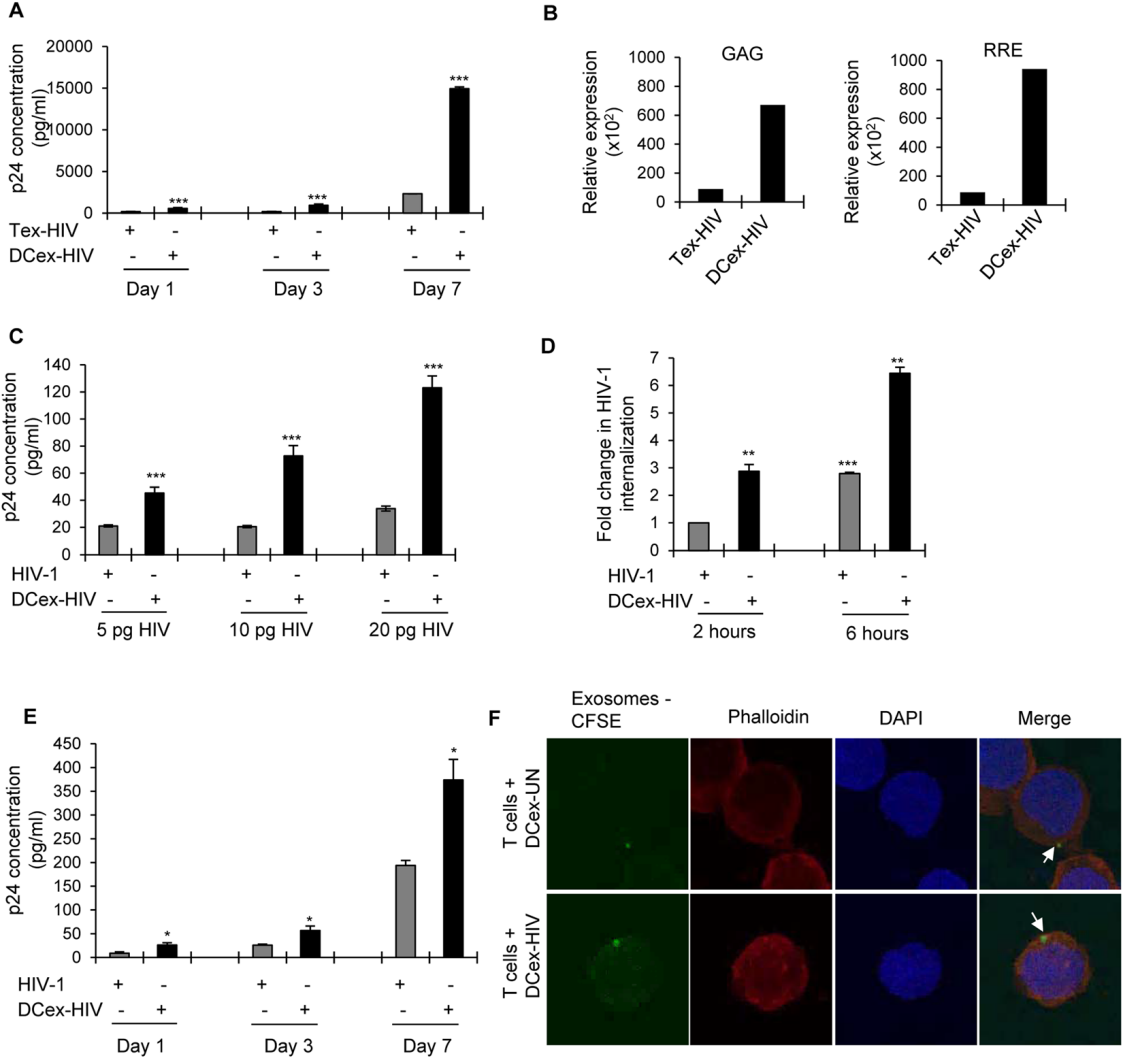
Analysis of HIV-1 infectivity of T cells mediated by exosomes derived from HIV-1 infected T cells and DCs. (**A**) CD63+ve exosomes isolated from HIV-1 infected T cells (Tex-HIV) or DCs (DCex-HIV) were cultured with T cells and cell supernatants were analyzed for HIV-1 p24 titer on days 1, 3, and 7 by using ELISA (***p ≤ 0.001, p-values for day 1 – 0.00066, day 3 – 0.00006, day 7 – 0.000147). (**B**) Total RNA was isolated from Tex-HIV and DCex-HIV incubated with T cells on day 7. Expression of HIV-1 genes GAG and RRE was assessed by qRT-PCR. (**C**) T cells were incubated with equal amounts (p24 titer) of CD63+ve DCex-HIV and cell free virus with indicated doses for 3 days and cell supernatants were analyzed for HIV-1 p24 titer using ELISA (***p ≤ 0.001, p-values for CD63+ve DCex-HIV 5 pg – 0.000714, 10 pg – 0.000294, 20 pg – 0.000062). (**D**) Bar diagram showing HIV-1 internalization in target T cells incubated with cell free HIV-1 or exosomes derived from HIV-1 infected DCs at indicated time points. Fold change was calculated by considering internalization in HIV-1 treated cells as 1 (**p ≤ 0.01, ***p ≤ 0.001, p-values for DCex-HIV 2 hours – 0.0089, 6 hours – 0.002099, HIV-1 6 hours – 0.000325). (**E**) T cells were incubated with equal amounts (p24 titer) of CD81+ve DCex-HIV and cell free virus for 7 days and cell supernatants were analyzed for HIV-1 p24 titer at day 1, 3 and 7 using ELISA (*p ≤ 0.05, p-values for DCex-HIV Day 1–0.0475, Day 3–0.0466, Day 7–0.0293. (**F**) Confocal images of exosomes internalization by T cells: DCex-UN and DCex-HIV were labelled with CFSE (green), incubated with T cells for 1 hour; cells were stained for Phalloidin (red) and analyzed by confocal microscopy (Arrow indicates exosomes). Results are representative of 3 independent experiments.

Previously, it has been shown that microvesicles derived from HIV-1 infected immature DCs and macrophages can facilitate HIV-1 trans-infection^13,31^. Hence, we analyzed the ability of exosomes derived from HIV-1 infected T cells and DCs to infect fresh T cells. To exclude cell free virions in the exosome preparations, we further isolated CD63 positive exosomes from the initial ultracentrifugation preparation by a validated immune affinity purification method using anti-CD63 antibody conjugated magnetic beads^45^. We then incubated T cells with CD63+ve exosomes derived from HIV-1 infected T cells or DCs and analyzed HIV-1 p24 titer in cells supernatants at days 1, 3 and 7 after exposures. We observed a 7–8 fold increase in HIV-1 p24 titer in T cells exposed to CD63+ve exosomes derived from HIV-1 infected DCs compared to those from HIV-1 infected T cells (Fig. 2A). However, the ability of the CD63+ve exosomes derived from HIV-1 infected T cells to transfer infection to fresh T cells ranged from no to minimal infection (Fig. 2A). We confirmed these results with qRT-PCR and noted a significant increase in HIV-1 replication in T cells exposed to exosomes derived from HIV-1 infected DCs compared to exosomes derived from HIV-1 infected T cells (Fig. 2B). Further, we compared the ability of exosomes derived from DCs with cell free HIV-1 virions to transfer infection by incubating T cells with equal amounts of p24; we observed a 4 fold increase in p24 titer in cells exposed to CD63+ve exosomes derived from HIV-1 infected DCs compared to cell free virions. These results indicated that exosomes derived from HIV-1 infected DCs can successfully spread the infection; moreover they are 4 fold more infective than cell free HIV-1 virions (Fig. 2C). Next, we compared exosome mediated internalization of HIV-1 with internalization of cell-free HIV-1 virus into target T cells by performing internalization assay. We observed a significant increase in viral internalization in T cells exposed to exosomes derived from infected DCs compared to T cells exposed to cell free HIV-1 virus at 2 to 6 hours post exposure (Fig. 2D). Since we observed enhanced expression of CD81 in DC exosomes, we immunopurified CD81+ve exosome population from total exosome preparation by using anti-CD81 antibody conjugated magnetic beads. Further, we performed infectivity assay by incubating T cells with equivalent amount of p24 concentration of CD81+ve exosomes derived from HIV-1 infected DCs and cell free HIV-1. We observed a significant increase in infectivity of T cells exposed to CD81+ve exosomes derived from HIV-1 infected DCs as compared to T cells exposed to cell free HIV-1 (Fig. 2E). We also analyzed the ability of exosomes derived from uninfected and HIV-1 infected DCs to be internalized by T cells by incubating the T cells with exosomes labelled with CFSE, a fluorescent cell staining dye. Confocal microscopic analysis revealed that exosomes derived from both uninfected and HIV-1 infected DCs were successfully internalized in T cells (Fig. 2F).

### Molecular composition of exosomes

**Figure 3:**
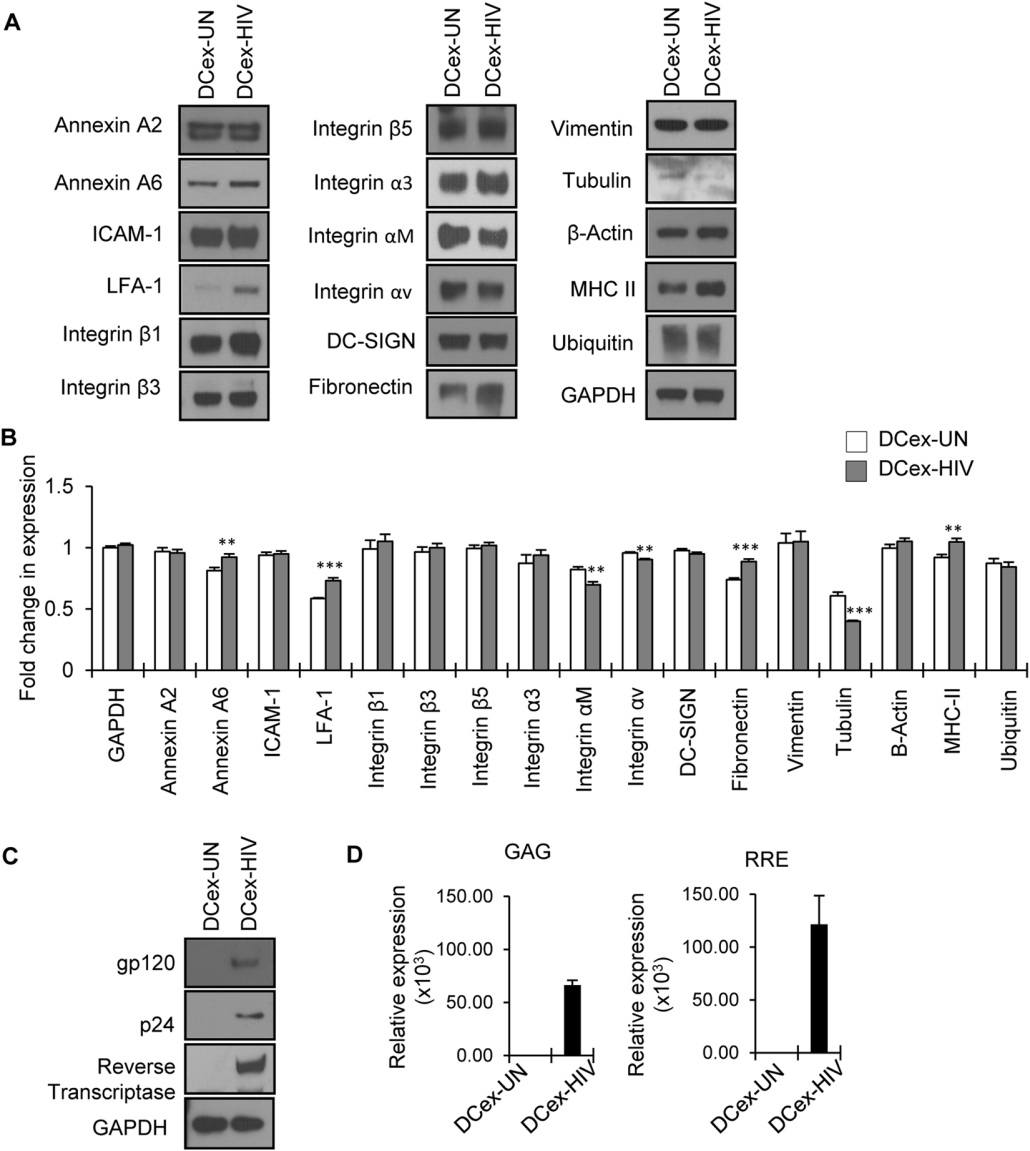
Molecular analysis of exosomes from uninfected and HIV-1 infected DCs (DCex-UN and DCex-HIV). (**A**) Representative Western blot images showing expression of indicated proteins in the lysates (10 µg) of exosomes derived from uninfected (DCex-UN) or HIV-1 infected (DCex-HIV) DCs. GAPDH served as a loading control. (**B**) Quantitative analysis of Western blot images of (**A**); the band intensity of each lane was determined by Image J software and pixel density was calculated. Fold change was determined by considering band intensity of GAPDH in untreated cells as 1 (**p ≤ 0.01, ***p ≤ 0.001, p-values for Annexin A6 – 0.00645, LFA-1 – 0.00047, Integrin αM – 0.00269, Integrin αv – 0.00112, Fibronectin – 0.00051, Tubulin – 0.00028, MHC-II – 0.00408. (**C**) Representative Western blot images showing expression of HIV-1 proteins in the lysates of exosomes derived from uninfected or HIV-1 infected DCs. GAPDH was used as a loading control. Full-length blots are presented in Supplementary Figure S3. (**D**) Total RNA was isolated from exosomes derived from uninfected (DCex-UN) or HIV-1 infected (DCex-HIV) DCs and analyzed for the expression HIV-1 genes by qRT-PCR. The data represents mean of triplicates ± SE from three independent experiments.

Exosomes have been shown to contain proteins, lipids and nucleic acids and their cargo may vary depending on the cell type and ambient conditions^24–26^. Hence, we analyzed the molecular composition of exosomes derived from uninfected and HIV-1 infected DCs. Western blot analysis revealed the expression of membrane trafficking/fusion proteins Annexin A2 and Annexin A6, adhesion molecules including ICAM-1, LFA-1, integrin β1, integrin β3, integrin β5, integrin α3, integrin αM and integrin αV and membrane proteins fibronectin and actin (Fig. 3A). Further, when we compared the expression pattern of these molecules, we observed increased expression of Annexin A6, ICAM-1, LFA-1, integrin β1, integrin α3 and fibronectin, while there was decreased expression of integrin αM and integrin αV in exosomes derived from HIV-1 infected DCs compared to exosomes derived from uninfected DCs (Fig. 3A and B). Antigen presenting cells (APCs), including dendritic cells, are known to secrete MHC-II bearing immunologically active exosomes^46^. Our analysis of exosomes derived from DCs also showed expression of MHC II molecules that was significantly enhanced in those derived from HIV-1 infected DCs (Fig. 3A and B). We also analyzed the expression of these molecules in total cell lysates of uninfected and HIV-1 infected DCs (Supplementary Figure S1). We observed increased expression of LFA-1, fibronectin and MHC II in exosomes derived from HIV-1 infected DCs compared total cell lysates of HIV-1 infected DCs. Further, we examined the presence of HIV-1 viral components in our preparation and observed structural components such as p24, gp120 and reverse transcriptase enzyme (Fig. 3C). We also tested for HIV-1 genome in total RNA isolated from exosome preparations by qRT-PCR, and observed the HIV gene elements GAG and RRE in exosomes derived from HIV-1 infected DCs (Fig. 3D).

### Exosomes derived from HIV-1 infected DCs transmitted infection via fibronectin and galectin-3

**Figure 4:**
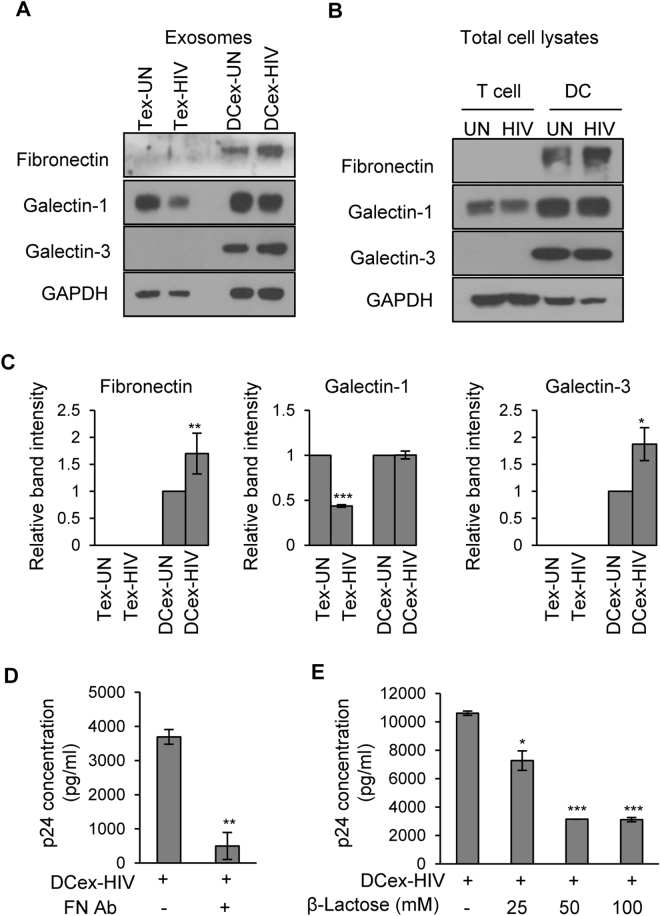
Exosomes derived from HIV-1 infected (DCex-HIV) DCs infected T cells via Fibronectin and Galectin. (**A** and **B**) Representative Western blot images showing the expression of fibronectin, galectin-1 and galectin-3 in lysates of exosomes derived from uninfected (Tex-UN) or HIV-1 infected (Tex-HIV) T cells, and uninfected (DCex-UN) or HIV-1 infected (DCex-HIV) DCs **(A)** and total cell lysates (**B**). GAPDH used as a loading control. Full-length blots are presented in Supplementary Figure S3. (**C**) Quantitative analysis of Western blot images of fibronectin, galectin-1 and galectin-3 of (**A**, exosomes). The band intensity of each lane was determined by Image J software and pixel density was calculated by normalizing to exosomes from uninfected cells. (*p ≤ 0.05, **p ≤ 0.01, ***p ≤ 0.001, p-values for Fibronectin – 0.00764, Galectin-1 – 0.0000003, Galectin-3–0.03248). (**D**) HIV-1 p24 titer in supernatants of T cells incubated with DCex-HIV in presence or absence of anti-fibronectin antibody for 7 days (**p ≤ 0.01, p-value – 0.009764). (**E**) HIV-1 p24 titer in supernatants of T cells incubated with DCex-HIV in presence of various concentrations (0–100 mM) of β-lactose for 7 days. The data represents mean of triplicates ± SE from three independent experiments (*p ≤ 0.05, ***p ≤ 0.001, p-values for 25 mM – 0.02144, 50 mM – 0.000201, 100 mM – 0.000402).

Since we observed a marked increase in the expression of fibronectin in exosomes derived from HIV-1 infected DCs, we further compared the expression pattern of this molecule in various exosomes. Interestingly, we did not observe fibronectin expression in exosomes derived from either uninfected or HIV-1 infected T cells, whereas exosomes derived from DCs showed high expression of fibronectin and its expression was significantly increased with HIV-1 infection (Fig. 4A).

Previous studies have shown that fibronectin and galectin-3 can enhance HIV-1 entry and infection up to 20 fold^31,47,48^. Hence, we analyzed the expression pattern of galectin-1 and -3 in these exosomes and observed galectin-1 in those derived from both HIV-1 infected or uninfected DCs and T cells. Exosomes derived from HIV-1 infected T cells showed a marked decrease in galectin-1 expression compared to those from uninfected T cells. Notably, exosomes derived from the T cells lacked expression of galectin-3, whereas DC derived exosomes showed enhanced expression of galectin-3. Further, expression of galectin-3 was significantly enhanced in exosomes derived from HIV-1 infected DCs compared to those from uninfected DCs (Fig. 4A and C). Next, we also analyzed expression of these molecules in total cell lysates of uninfected and HIV-1 infected T cells and DCs. Intriguingly, T cell total cell lysates lacked the expression of fibronectin and galectin-3, whereas DC total cell lysates showed high expression of these molecules (Fig. 4B).

To investigate the role of fibronectin and galectin-3 in DC derived exosome mediated transmission of HIV-1, we incubated T cells with these exosomes with or without anti-fibronectin antibodies for 7 days and quantitated the p24 titer in cell supernatant. We observed that anti-fibronectin antibodies significantly blocked exosome mediated HIV-1 transmission to T cells (Fig. 4D). Furthermore, we studied DC exosome mediated HIV-1 transmission to T cells in the presence of various concentrations (0–100 mM) of β-lactose, a natural antagonist of galactin-3^48,49^, and observed that β-lactose significantly blocked virus transmission (Fig. 4E). These results indicate that fibronectin and galectin-3 may be involved in HIV-1 transmission mediated by exosomes derived from HIV-1 infected DCs.

### DC derived exosomes induced activation of p38/Stat pathway and adhesion molecules

**Figure 5:**
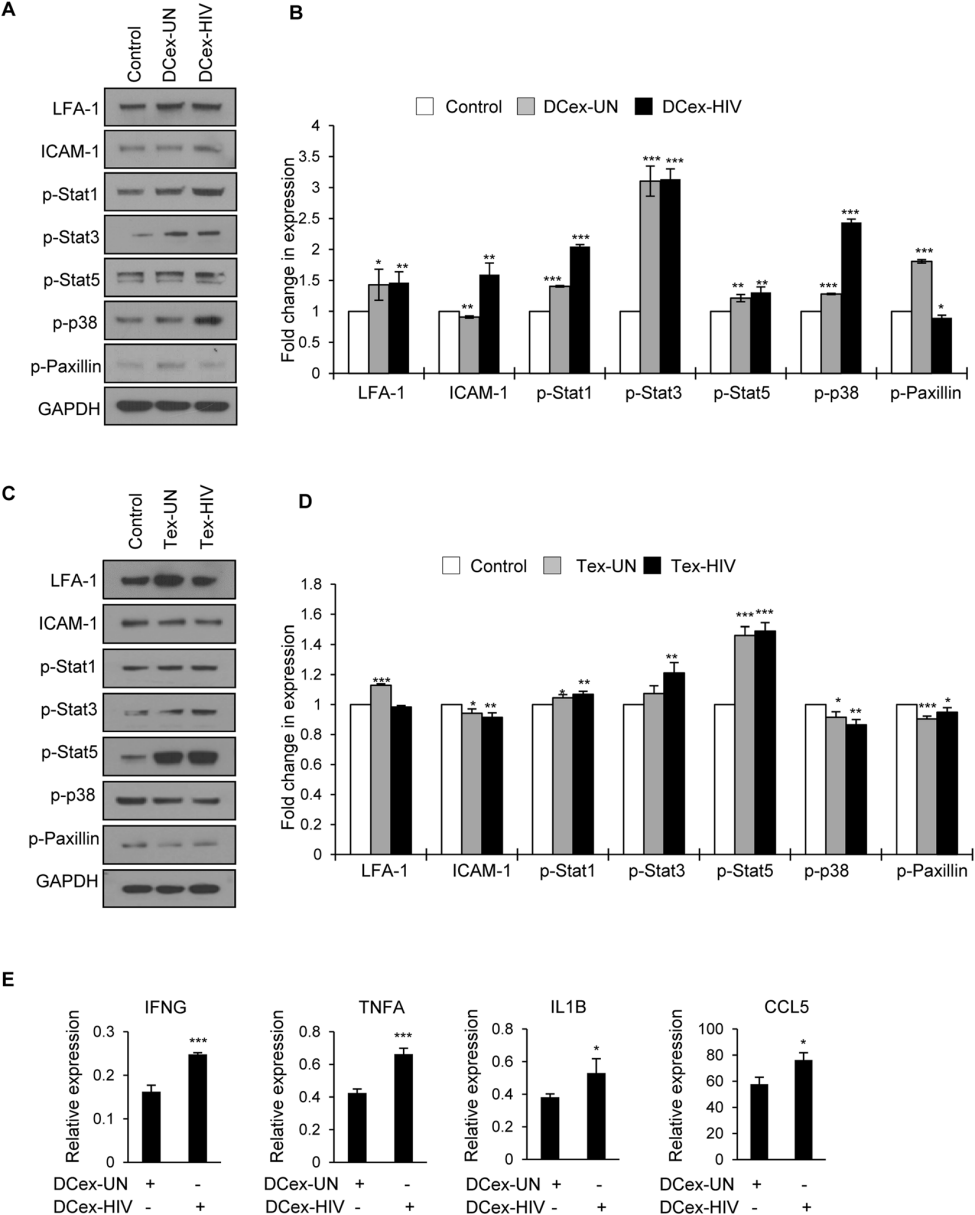
Exosomes derived from HIV-1 infected DCs activated p38/STAT signaling and induced expression of pro-inflammatory cytokines in T cells. (**A**) Representative Western blot images of indicated signaling molecules in T cells incubated with or without (control) exosomes derived from uninfected (DCex-UN) or HIV-1 infected (DCex-HIV) DCs for 24 hours. GAPDH was used as a loading control. Full-length blots are presented in Supplementary Figure S3. (**B**) Quantitative analysis of Western blot images of (**A**); the band intensity of each lane was determined by Image J software and pixel density was calculated by normalizing to control. (*p ≤ 0.05, **p ≤ 0.01, ***p ≤ 0.001, p-values for LFA-1 – 0.04053, 0.009804; ICAM-1 – 0.00135, 0.00519; p-Stat1 – 0.0000008, 0.0000006; p-Stat3 – 0.000114, 0.000023; p-Stat5 – 0.0032, 0.00365; p-p38 – 0.0000015, 0.0000014; p-Paxillin – 0.000001, 0.0142). (**C**) Representative Western blot images of indicated signaling molecules in T cells incubated with or without (control) exosomes derived from uninfected (Tex-UN) or HIV-1 infected (Tex-HIV) T cells for 24 hours. GAPDH was used as a loading control. Full-length blots are presented in Supplementary Figure S3. (**D**) Quantitative analysis of Western blot images of (**C**); the band intensity of each lane was determined by Image J software and pixel density was calculated by normalizing to control (*p ≤ 0.05, **p ≤ 0.01, ***p ≤ 0.001, p-values for LFA-1 – 0.00002; ICAM-1 – 0.025, 0.0086; p-Stat1 – 0.0222, 0.0047; p-Stat3 – 0.00633; p-Stat5 – 0.00015, 0.000105; p-p38 – 0.016, 0.0028; p-Paxillin – 0.00099, 0.0434). (**E**) qRT-PCR for IFNG, TNFA, IL1B, and CCL5 in T cells incubated with or without DCex-UN or DCex-HIV for 3 days. Relative gene expression of cytokines in T cells incubated with DCex-UN or DCex-HIV. The data represents mean of triplicates ± SE from three independent experiments (*p ≤ 0.05, **p ≤ 0.01, ***p ≤ 0.001, p-values for IFNG – 0.000655, TNFA – 0.00073, IL1B – 0.04711, CCL5 – 0.01419).

Exosomes have been shown to activate various signaling molecules in the recipient cells including the MAP kinases, NF-kB and focal adhesion molecules^50,51^. Hence, we studied the signaling molecules activated in T cells upon exposure to DC exosomes. We observed increased phosphorylation of MAP kinase p38, Stat1, Stat3 and Stat5. These results indicate that exosomes derived from DCs may activate p38/Stat pathways which can enhance the production of pro-inflammatory cytokines. Moreover, we observed increased expression of LFA-1 and ICAM-1 and increased phosphorylation of Paxillin in T cells exposed to DC exosomes (Fig. 5A and B). These molecules are known to play important roles in T cell adhesion and migration. Next, we studied the activation these signaling molecules in T cells exposed to exosomes derived from uninfected and HIV-1 infected T cells. As observed in T cells exposed to DC exosomes, we found enhanced expression of LFA-1 and increased phosphorylation of Stat1, Stat3 and Stat5, whereas a significant decrease in phosphorylation of p38 and paxillin in these cells (Fig. 5C and D).

### Exosomes derived from HIV-1 infected cells stimulated the production of pro-inflammatory cytokines

Recent studies have shown that exosomes derived from HIV-1 infected primary macrophages stimulated the production of pro-inflammatory cytokines in T cells^52^. Hence, we tested a panel of cytokines from T cells incubated with exosomes derived from uninfected DCs or HIV-1 infected DCs by using qRT-PCR. We observed a significant increase in the gene expression of IFN-γ, TNF-α and IL-1β in T cells exposed to exosomes derived from infected DCs compared to those from uninfected DCs. Moreover, we observed a significant increase in the expression of RANTES (CCL5) in T cells exposed to exosomes derived from HIV-1 infected DCs (Fig. 5E). These results indicate that exosomes derived from HIV-1 infected DCs can stimulate the production of pro-inflammatory cytokines in T cells.

### Differences in the exosome biogenesis machinery between T cells and DCs

**Figure 6:**
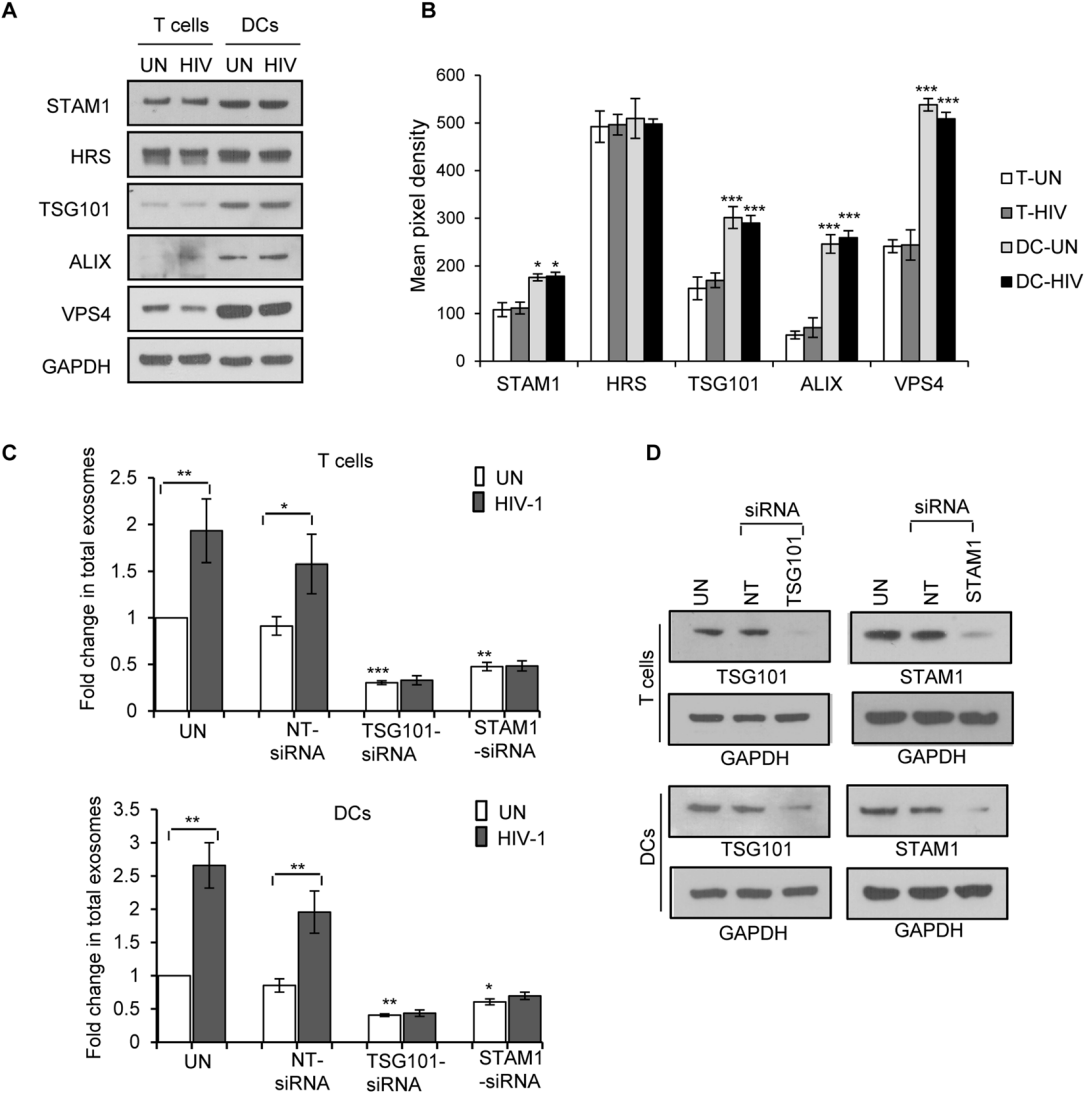
Analysis of molecules involved in exosome biosynthesis in T cells and DCs. (**A**) Representative Western blot images of indicated molecules in total cell lysates from uninfected (UN) and HIV-1 infected (HIV) T cells and DCs after 3 days. GAPDH used as loading control. (**B**) Quantitative analysis of Western blot images of (**A**); the band intensity of each lane was determined by Image J software and pixel density was calculated. The data represents mean of triplicates ± SE from three independent experiments. (*p ≤ 0.05, **p ≤ 0.01, ***p ≤ 0.001, p-values for STAM 1- 0.019, 0.03; TSG101-0.00018, 0.00013, ALIX-0.00026, 0.001, VPS4-0.00012, 0.00031). (**C**) Fold change in total exosomes in siRNA-TSG101 or siRNA-STAM1 transfected T cells and DCs. Fold change was calculated by considering exosome release from untreated and uninfected cells as 1 (unfilled bars-uninfected cells; filled bars- HIV-1 infected cells). The data represents mean of three independent experiments (*p ≤ 0.05, **p ≤ 0.01, ***p ≤ 0.001, p-values of T cells are 0.0073; 0.0221; 0.00098; 0.005697262. p-values of DCs are 0.0010; 0.00462; 0.0016; 0.023). (**D**) Representative Western blot images showing the expression of TSG101 and STAM1 in untreated (UN), control siRNA (NT) and siRNA-TSG101 or siRNA-STAM1 transfected T cells or DCs. GAPDH used as loading control. Full-length blots are presented in Supplementary Figure S3.

The mechanisms involved in exosome biogenesis involve a complex multi-stage process with various molecules contributing to intraluminal vesicle (ILV) formation, cargo recruitment and exosome release^17^. We observed considerable differences in number, composition, morphology and biological functions of exosomes derived from T cells and DCs and thus examined the key molecules involved in these processes in immune cells. Endosomal Sorting Complex Required for Transport (ESCRT) family members play a role in the initial steps of exosome formation^11,46,53^. We found a significant decrease in the expression of Signal Transducing Adaptor Molecule 1 (STAM1), a component of ESCRT-0 complex, in T cells compared to DCs^54^. However, we did not observe significant differences in expression of Hepatocyte growth factor-regulated tyrosine kinase substrate (HRS), also a component of ESCRT complex^54^, between these cell types. In addition, expression of TSG101, an ESCRT-I associated protein, and Alix and VPS4, ESCRT-III associated proteins^11,17^, also were significantly reduced in T cells compared to DCs (Fig. 6A and B). Next, to analyze the role of TSG101 and STAM1 in the biogenesis of exosomes in HIV-1 infected or uninfected T cells and DCs, we knocked down TSG101 or STAM1 in these cell types by siRNA technique. We then quantitated total exosomes from uninfected or HIV-1 infected siRNA-TSG101 or siRNA-STAM1 transfected T cells and DCs. We found that exosome release was significantly inhibited in TSG101 and STAM1 knocked down cells compared to control cells (Fig. 6C). We confirmed the knockdown of expression of TSG101 and STAM1 in both T cells and DCs by Western blot analysis (Fig. 6D). These results suggest that STAM1 and TSG101 may play a key role in the production of exosomes which can facilitate cell-to-cell HIV-1 transmission.

## Discussion

Although exosomes were first described almost 30 years ago, their molecular characterization and biological functions are not yet well established. Recent research indicates a role for exosomes in cell-cell communication, regulation of immune response, and alteration of the cellular microenvironment^17,28,41,55^. Exosomes have also been implicated in progression of various pathological conditions such as spread of viral infection, tumor metastasis, inflammation, and certain neurological disorders^9,11,56,57^.

The ‘Trojan exosome hypothesis’ initially proposed by Gould *et al*., suggested the role of exosomes in retroviral infection. In this model, they theorized that retroviruses may exploit host intracellular vesicle trafficking pathways and biogenesis with release of extracellular vesicles or exosomes. These exosomes would possess a unique composition of both retroviral and host molecules and the ability to infect cells independently of envelope protein-receptor interactions^58^. Subsequently, Wiley *et al*. demonstrated that HIV-1 particles captured by DCs can be transmitted to T cells via secreted extracellular vesicles without *de novo* infection^13^. Further, exosomes and MVBs released from macrophages may facilitate productive HIV-1 infection^31^. In our study, we characterized exosomes derived from HIV-1 infected and uninfected T cells and DCs and addressed their possible biological functions. Though an exclusive marker for exosomes is yet to be identified, Tetraspanin CD63, known to associate with membranes of intracellular vesicles, is considered as a ubiquitous marker for exosomes^59^. Upon analysis, our exosome preparations from both T cells and DCs were positive for CD63. Interestingly, two other exosomal markers CD9 and CD81, both tetraspanin transmembrane proteins, were found only in exosomes derived from DCs, and were absent in exosomes from T cells. Previous studies have shown the presence of HSP70 in exosomes released from various cell types; its level is elevated in cellular stress conditions and during infection^60^. We observed HSP70 in exosome preparations from both T cells and DCs and significantly elevated levels in exosomes derived from HIV-1 infected cells. Intriguingly, our electron microscopic analysis revealed a different morphological pattern between exosomes derived from T cells and DCs. T cell exosomes showed uniform circular vesicles of 20–100 nm diameter with low electron densities, while exosomes from DCs were more pleomorphic with sizes ranging from 30–200 nm in diameter and exhibited high electron density at the center. Further, the numbers of exosomes released from HIV-1 infected T cells and DCs was significantly increased as compared to that from uninfected cells. Previous studies have shown that HIV-1 subverts host intracellular vesicle trafficking pathways to avoid its degradation and enhance its dissemination. This may have contributed in enhanced biogenesis of exosomes in HIV-1 infected cells^13^. Moreover, recent study by Edger *et al*.^61^ has demonstrated that tetherin (BST-2) restricts the release of exosomes by anchoring them to the cell surface. Hence, HIV-1 induced degradation of tetherin may result in enhanced release of exosomes into the extracellular milieu.

Several viruses, including HCV, HGV, HTLV and HIV-1, can be transferred to cells via exosomes^9,13,30,31^. These exosomes were shown to contain viral genome, peptides and miRNA along with host cellular cargo^9,11,28^. Studies have also indicated that exosomes may harbor virions or an entire viral genome^9,11,28^. While it has been reported that exosomes derived from HIV-1 infected cells can transmit infection, the characteristics of these exosomes, specifically their molecular composition and mechanisms through which they transfer the infection, are not well understood. In our study, in order to analyze HIV-1 transmission by exosomes derived from HIV-1 infected T cells and DCs, we further purified CD63+ve exosomes from our total exosome preparation to avoid any cell free HIV-1 viral contamination. Our functional analysis of CD63+ve exosomes revealed that exosomes derived from HIV-1 infected DCs successfully transmitted infection to T cells and induced 4–5 fold increased HIV-1 replication compared to cell free HIV-1 virus. Moreover, exosomes derived from HIV-1 infected T cells were at best weakly infective and induced low viral replication in T cells.

Molecular composition of exosomes derived from various cell types has been studied extensively by various techniques^25,28,31^. Consistent with previous studies, we observed the presence of adhesion molecules, including ICAM-1, LFA-1, and integrins, and cytoskeletal proteins such as actin and fibronectin^25,28,31^. Intriguingly, we found expression of fibronectin and galectin-3 exclusively in exosomes derived from DCs. Further their expression was significantly enhanced in exosomes derived from HIV-1 infected DCs. Fibronectin has been shown to enhance HIV-1 virus stability and its infectivity to CD4+ T cells^62,63^. Moreover, it has also been shown that fibronectin and its endogenous family of galectin ligands significantly enhanced HIV-1 entry and infection in permissible cells^47,48,62,63^. Recent studies have revealed that surface bound fibronectin mediates the exosome-cell interactions and antibodies specific for the Hep-II heparin binding domain of fibronectin can block such interaction^64^. In our study, we also observed anti-fibronectin antibodies significantly blocked DC exosome mediated HIV-1 infection of T cells. Galectins are host soluble β-galactoside-binding lectins which possess conserved peptide sequence elements in the carbohydrate recognition domain^48,65^. Galectin-1 is known to interact with gp120 and facilitate rapid HIV-1 infection of susceptible cells^48,65^. Further, the complex glycans on gp120 have shown specificity to galectin-1 but not to galectin-3^48,65^. In our study, β-lactose, an antagonist of galectins, significantly blocked DC exosome mediated HIV-1 infection of T cells. These results indicate that fibronectin and galectin may play significant roles in the interaction between T cells and exosomes derived from DCs and facilitate HIV-1 transmission. Moreover, we detected viral molecules including gp120, p24, reverse transcriptase, and HIV-1 RNA in exosomes derived from HIV-1 infected DCs.

Increasing evidence suggests that exosomes derived from APCs also may mediate antigen presentation as they contain MHC complex class I and II and T cell-co-stimulatory molecules^28,46^. We identified MHC II in exosomes derived from DCs, suggesting a role in antigen presentation and regulation of immune response. This further confirms previous findings by Admyre *et al.* ^18^, who demonstrated that DC exosomes containing viral antigens can stimulate T lymphocytes *in vitro* without the presence of DCs. Recently, it has been shown that exosomes released from mature DCs induced pro-inflammatory response in intestinal epithelial cells^52^. Our analysis of cytokines in T cells exposed to exosomes derived from HIV-1 infected DCs revealed increased gene expression of pro-inflammatory cytokines, specifically IFN-γ, TNF-α IL-1β and CCL5 (RANTES).

Since we observed distinctive differences in morphology, molecular composition and biological functions between exosomes derived from DCs and T cells, we hypothesize that there may be differences in exosome biogenesis machinery between these two cell types. Though the precise mechanisms involved in exosome biogenesis, cargo recruitment and its release are not yet fully defined, ESCRT complexes are known to play key roles in exosome biogenesis^17,46,53^. The ESCRT-0 complex may regulate ubiquitin dependent cargo clustering. ESCRT-I and ESCRT-II complexes are known to induce bud formation and ESCRT-III participates in vesicle scission and release^17,46,53^. In addition, ESCRT complex associated molecules such as Alix, HRS and TSG101 have shown to be involved in exosome release^17,46,53,54^. Our analysis revealed low expression of Alix, HRS, TSG101 and STAM1 in T cells compared to DCs, indicating that T cells might have different exosome biogenesis machinery. Such differences in expression of these molecules may account for functional and morphological properties between exosomes derived from these two cell types.

Our study for the first time demonstrates distinctive biogenesis and morphological and functional characteristics between exosomes derived from T cells and DCs and their role in cell-to-cell transmission of HIV-1. Further, we show exosomes derived from HIV-1 infected DCs can transmit HIV-1 to T cells and facilitate robust infection, compared to cell free virus, and further stimulate pro-inflammatory cytokine release from T cells. Understanding the role of DC derived exosomes in HIV-1 pathobiology may significantly contribute to development of therapeutic strategies to limit HIV-1 infection.

## Methods

### Cells, HIV-1 and constructs

Buffy coats were obtained from the Blood Transfusion Service, Massachusetts General Hospital, Boston, MA, in compliance with the Beth Israel Deaconess Medical Center Committee on Clinical Investigations (CCI) protocol #2008-P-000418/5. Buffy coats were provided at this institution for research purposes; therefore, no informed consent was further needed. In addition, buffy coats were provided without identifiers. This study was approved by Beth Israel Deaconess Medical Center’s CCI, Institutional Review Board, and Privacy Board appointed to review research involving human subjects. The experimental procedures were carried out in strict accordance with approved guidelines.

Human peripheral blood mononuclear cells were isolated from buffy coats and monocytes were isolated using a positive selection kit per manufacturer’s protocol (STEMCELL Technologies, Inc.). Monocyte derived dendritic cells (hereafter referred as DCs) were prepared and cultured as previously described^66^. Autologous T cells from human peripheral blood mononuclear cells, activated with PHA-L (1 μg/ml) and maintained in complete culture medium supplemented with IL-2 (10 ng/ml) (PeproTech, Rocky Hill, NJ) at 2 × 10^6^ cells/ml. Purity of these T cells was analyzed using CD3 and CD4 staining and flow cytometry.

HIV-1 BaL was obtained from the NIH AIDS Research and Reference Reagent Program, National Institute of Allergy and Infectious Diseases, NIH^67^. To prepare HIV-1 stocks, PBMC derived T cells were cultured with HIV-1 BaL for 7 days. Fresh T cells, suspended at 1 × 10^6^ cells/ml were added at day 7. At day 14 after initial viral inoculation, the supernatant was harvested and stored at −80 °C. p24 viral antigen in the supernatants was quantified by ELISA (Zeptometrix Corporation, Buffalo, NY).

### Antibodies and reagents

Annexin A2, DC-SIGN, ICAM-1, Integrin β1, Integrin β3, Integrin β5, HRS, STAM1, VPS4, p-p38, p-Paxillin, p-Stat1, p-Stat3, p-Stat5, Ubiquitin, Vimentin, and β-Actin antibodies were obtained from Cell Signaling Technology (Danvers, MA). CD63, CD9, CD81, HSP70, Integrin αM, Integrin α3, Integrin α5, Annexin A6, TSG101, Alix, and GAPDH antibodies were obtained from Santa Cruz Biotechnology, Inc. (Santa Cruz, CA). LFA-1 antibody was obtained from BD Biosciences; MHC II and Fibronectin antibodies were obtained from Abcam (Cambridge, MA), and Tubulin antibody was obtained from Sigma-Aldrich (St. Louis, MO). HIV-1 p24 gag and HIV-1 gp120 antibodies were obtained from ABL (Rockville, MD) while the HIV-1 Reverse Transcriptase antibody was obtained from Invitrogen (Carlsbad, CA). APC anti-human CD63 antibody and APC Mouse IgG1, κ Isotype Ctrl (FC) were obtained from Biolegend (San Diego, CA). Exosome-depleted FBS was obtained from System Biosciences (Palo Alto, CA). β-lactose was obtained from Sigma-Aldrich (St. Louis, MO).

### Exosome isolation

For exosome preparations, both T cells and DCs (2 × 10^6^ cells/ml) from three donors were infected with HIV-1 BaL (10 ng/ml of HIV-1 p24) in an exosome depleted medium. After 3 days, we quantitated the p24 titer in cell supernatants of both T cells and DCs. Exosomes were isolated from cell supernatants by a combination of centrifugation and filtration: 700 × g to remove cells and debris, filtering the supernatants on 0.45 µm pore filters, followed by ultracentrifugation at 100,000 × g (Beckman Optima TLX, rotar- TLA 100.4) and washing with 0.2 µm filtered 1X PBS by ultracentrifugation at 100,000 × g. Next, CD63+ve exosomes or CD81+ve exosomes were purified using Exo-Flow^™^ Exsosome Purification kits (System biosciences, Mountain View, CA) or Exosome-Human CD63 Isolation/Detection Reagent from Invitrogen (Carlsbad, CA) as per manufacturer’s protocol. In each exosome preparation, the concentration of total proteins was quantified by Bradford assay (Bio-Rad Laboratories, Hercules, CA). Exosomes were quantified by using EXOCET Assay system (System biosciences). For experiments with RNA, pellets were lysed with RNA lysis buffer to isolate RNA.

### Western blotting

Western blotting was performed as previously described^66^. Briefly, exosomes or uninfected and HIV-1 infected T cells and DCs or T cells co-cultured with exosomes (after incubation period) were collected in cell lysis buffer, protein lysates were separated on NuPAGE precast gels (Life Technologies Corp.), transferred to 0.45 μm nitrocellulose membranes (Bio-Rad Laboratories, Hercules, CA), and probed with appropriate primary antibodies followed by incubation with their respective secondary antibodies. Proteins were visualized with Western Lightning Plus ECL Substrate (PerkinElmer, Waltham, MA).

### Electron Microscopy

Isolated exosomes were fixed with 4% PFA by resuspending the pellet in the fixative prior to negative staining. Nickel grids with 200 mesh, formvar, carbon coating, and freshly glow discharged were used for the negative staining of matrix vesicles. Each grid was placed on a 20 µl droplet of sample for 5 minutes. The grid was washed on three droplets of water and then negatively stained with phosphotungstic acid for 20 seconds or 2% Uranyl Acetate for 1 minute. Excess liquid was wicked off the grids with Whatman 50 filter paper. Exosome samples were analyzed on JEOL JEM-1400 transmission electron microscope (JEOL USA Inc., Peabody, MA). The number of exosomes in each size range (nm) was quantitated by counting in 10 different fields of electron microscope images.

Immunogold Labelling: Exosome samples were adsorbed to a carbon coated grid that was made hydrophilic by a 30 second exposure to a glow discharge, then blocked with 1% BSA and incubated with anti-human CD63 antibodies for 30 minutes. After washing with PBS, grids were treated with rabbit anti mouse bridging antibody followed by Protein A-gold (10 nm) in 1% BSA. The samples were stained with 0.75% uranyl formate for 30 seconds. After removing the excess uranyl formate, the grids were examined in a TecnaiG² Spirit BioTWIN and images were recorded with an AMT 2k CCD camera.

### Confocal microscopy

Exosome pellets from uninfected and HIV-1 infected DCs resuspended in PBS (100 ng total protein content) were labeled with 5 μM CFSE for 30 minutes at 37 °C, followed by washing with 1X PBS to remove unbound dye at 100,000 × g. The labeled exosomes were incubated with T cells for 1 hour. Then T cells were fixed in 4% paraformaldehyde and blocked with 5% normal goat serum in PBS/Triton × 100 for 1 hour. Cells were then incubated with Rhodamine-phalloidin (Molecular Probes) for 2 hours. Subsequently, cells were washed thrice with PBS, and slides were mounted using Prolong Gold antifade with DAPI (4′,6-diamidino-2-phenylindole; Invitrogen). Slides were examined under a Zeiss 880 Meta confocal microscope (Carl Zeiss Microimaging, LLC, Thornwood, NY), and images were acquired using ZEN2 software (Carl Zeiss). Figures were made using Adobe Photoshop CS4 software (Adobe Systems, San Jose, CA).

### Exosome infectivity in T cells

T cells (1 × 10^6^/ml) were infected either with 1 ug of total protein of CD63+ve exosomes derived from uninfected and HIV-1 infected T cells and DCs or with 5–20 pg p24 of CD63+ve exosomes or CD81+ve exosomes derived from HIV-1 infected DCs and HIV-1 BaL. T cells were incubated for 7 days at 37 °C with or without anti-fibronectin antibodies (1:100) or β-lactose (25, 50 and 100 mM). Supernatants were harvested on days 1, 3 and 7, and p24 concentrations were quantified by ELISA.

### HIV-1 internalization assay

T cells (1.0 × 10^6^ cells/ml) were incubated with HIV-1 (10 ng/ml, p24) or exosomes derived from HIV-1 infected DCs for 2–6 hours at 37 °C, unbound virus particles were removed by washing the cells three times in 1X PBS. The cells were then incubated with 0.05% trypsin for 5 minutes to remove surface bound viral particles. DCs were lysed with cell lysis buffer (RIPA containing 1% Triton X-100). Viral binding was estimated by quantitating the p24 in the cell lysates by ELISA. Total cell protein was estimated and all samples were normalized for protein content.

### Quantitative RT-PCR

RNA was isolated from exosomes and T cells incubated with DC exosomes by Quick-RNA MiniPrep isolation kit according to the manufacturer’s instructions (Zymo Research, Irvine, CA). DNase treatment was performed using TURBO DNA-free kit (Ambion RNA, Carlsbad, CA). 0.4ug and 1ug of exosomal RNA and T cell RNA respectively were used to prepare cDNA using iSCRIPT cDNA synthesis kit (Bio-Rad, Hercules, CA). qRT-PCR was done in triplicate for each sample with SYBR green based SsoFast EvaGreen Supermix (Bio-Rad Laboratories, Hercules, CA) using 50 ng cDNA. Gene expression was normalized to TATA-box binding protein (TBP) and relative expression was calculated using 2−ΔCt method. Specificity of the primer sets was confirmed by melting curve analysis.

Primer sequences are IFNG_F: TCCTTTGGACCTGATCAGCTTG, IFNG_R: AACCCAAAACGATGCAGAGC; CCL5_F: TGCTGCTTTGCCTACATTGC, CCL5_R: ACACACTTGGCGGTTCTTTC; GAG_F: TTGGTCCAAAATGCGAACCC, GAG_R: ACTTGGCTCATTGCTTCAGC; RRE_F: TGGGCAAGTTTGTGGAATTGG, RRE_R: ACCTACCAAGCCTCCTACTATC; IL1A_F: AGCTGATGGCCCTAAACAGATG, IL1A_R: TTGTCCATGGCCACAACAAC; TNFA_F: AAGTGCTGGCAACCACTAAG, TNFA_R: TCAAGTCCTGCAGCATTCTG; TBP_F: TCACTGTTTCTTGGCGTGTG, TBP_R: TGGCAAACCAGAAACCCTTG.

### siRNA-mediated knockdown of TSG101 and STAM1

Small interference RNA-mediated knockdown of TSG101 or STAM1 was performed using siRNA-TSG101 or siRNA-STAM1 (Santa Cruz Biotechnology). A non-targeting siRNA (Qiagen, Valencia, CA) was used as the negative control. siRNAs were transfected into T cells and DCs by nucleofection (Lonza Wakersville Inc, Wakersville, MD) per manufacturer’s instructions.

### Statistical Analysis

Differences between groups were calculated using a standard 2-tailed Student’s t-test. p-values ≤ 0.05 were considered statistically significant.

## Electronic supplementary material

Supplementary Figures
