# Supplementary material for: Exosomes Derived from HIV-1 Infected DCs Mediate Viral trans-Infection via Fibronectin and Galectin-3
Source: Sci Rep. 2017 Nov 1;7:14787. doi: 10.1038/s41598-017-14817-8 (PMC5665889; doi:10.1038/s41598-017-14817-8)

Exosomes Derived from HIV-1 Infected DCs Mediate Viral trans-Infection via Fibronectin and Galectin-3

Rutuja Kulkarni, Anil Prasad

Supplementary Figure S1

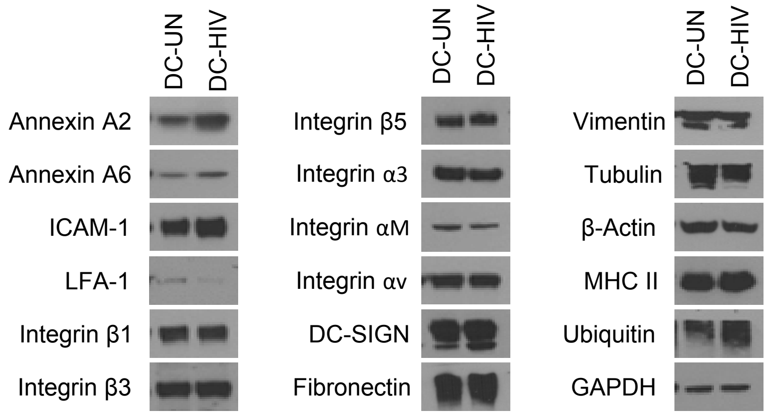

Supplementary Figure S1: Representative Western blot images showing expression of indicated proteins in the total cell lysates of uninfected (DC-UN) DCs or HIV-1 infected (DC-HIV) DCs. GAPDH served as a loading control.

Supplementary Figure S2

A B

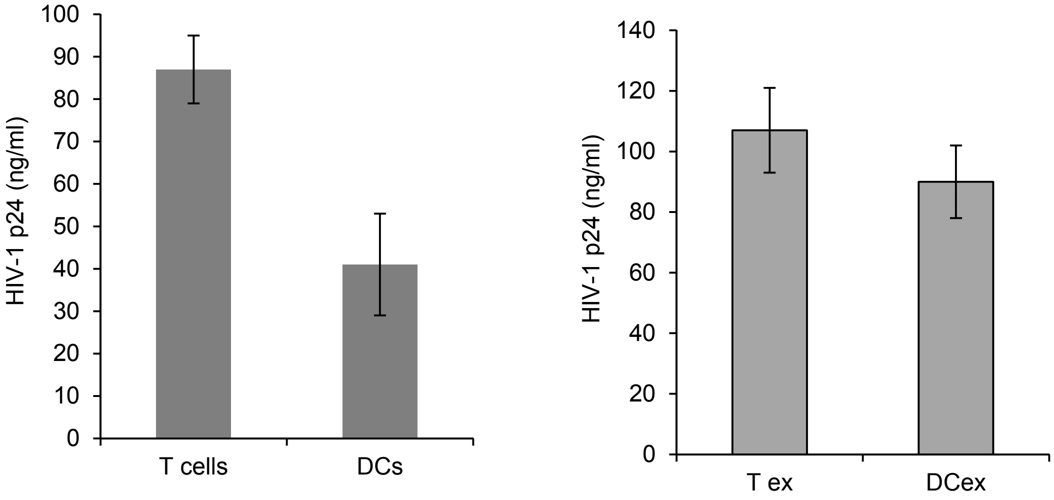

Supplementary Figure S2: (A) HIV-1 p24 titer in T cells and DCs: T cells and DCs (2x10^6 cells/ml) incubated with HIV-1 BaL (10ng/ml) for 3 days. HIV-1 p24 concentration was quantitated in cell Suparenant by using HIV-1 p24 ELISA. **(B) HIV-1 p24 titer in exosomes**: Exosomes were isolated from Cell supernatant of above mentioned cells. HIV-1 p24 concentration was quantitated in cell lysates by using HIV-1 p24 ELISA.

Supplementary Figure S3

Supplementary Fig. S3A: Full-length blots for Figure 1A.

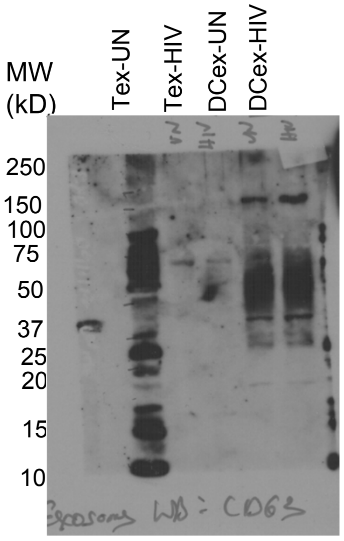

CD63

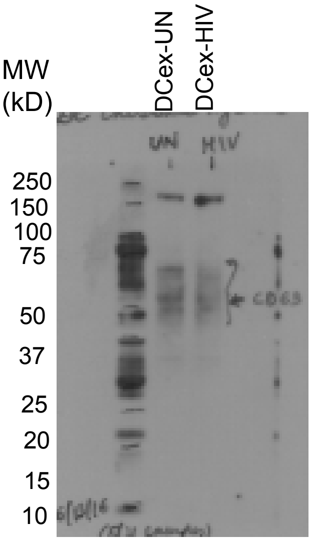

CD63

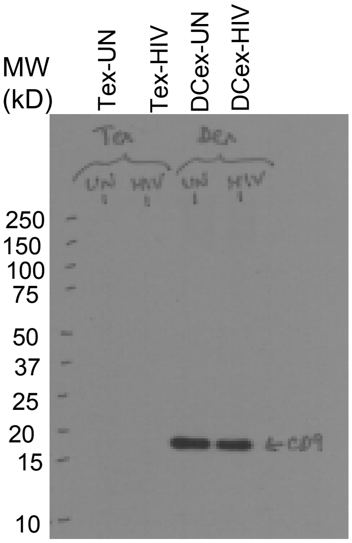

CD9

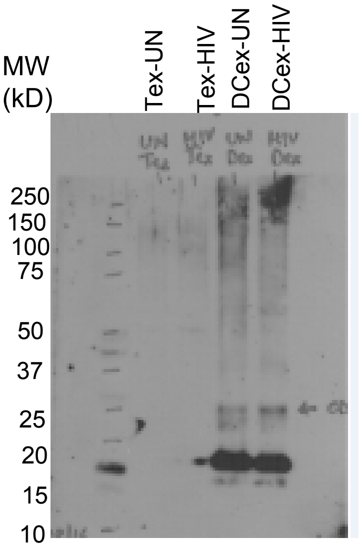

CD81

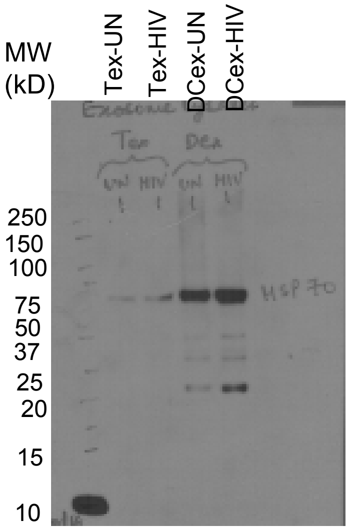

HSP70

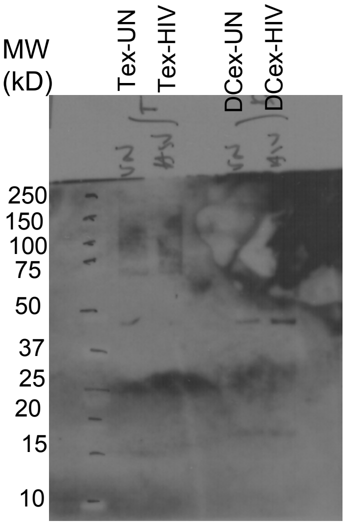

TSG101

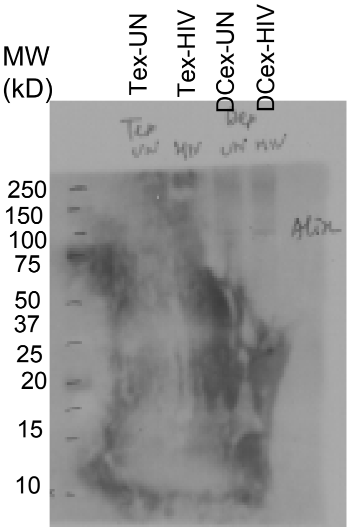

Alix

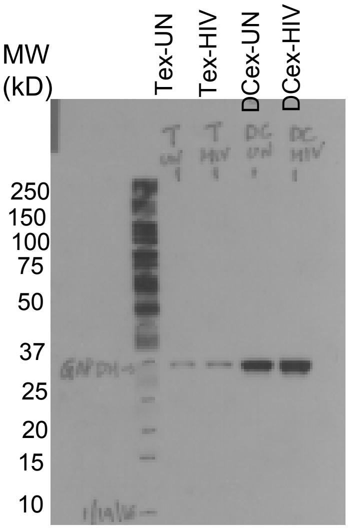

GAPDH

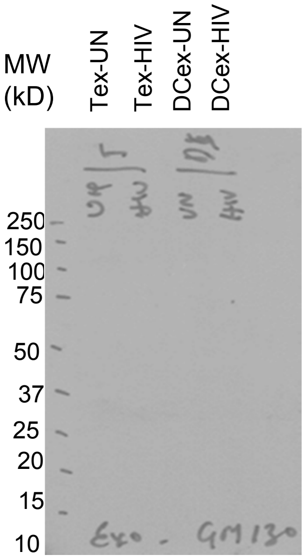

GM130

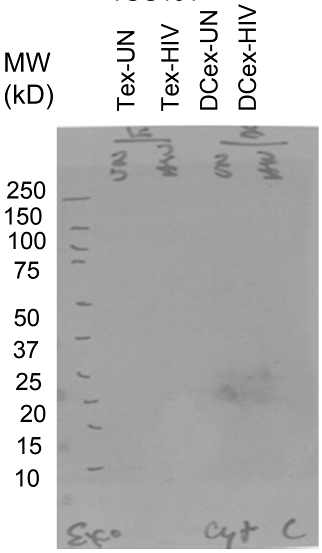

Cytochrome C

Supplementary Fig. S3B: Full-length blots for Figure 1B.

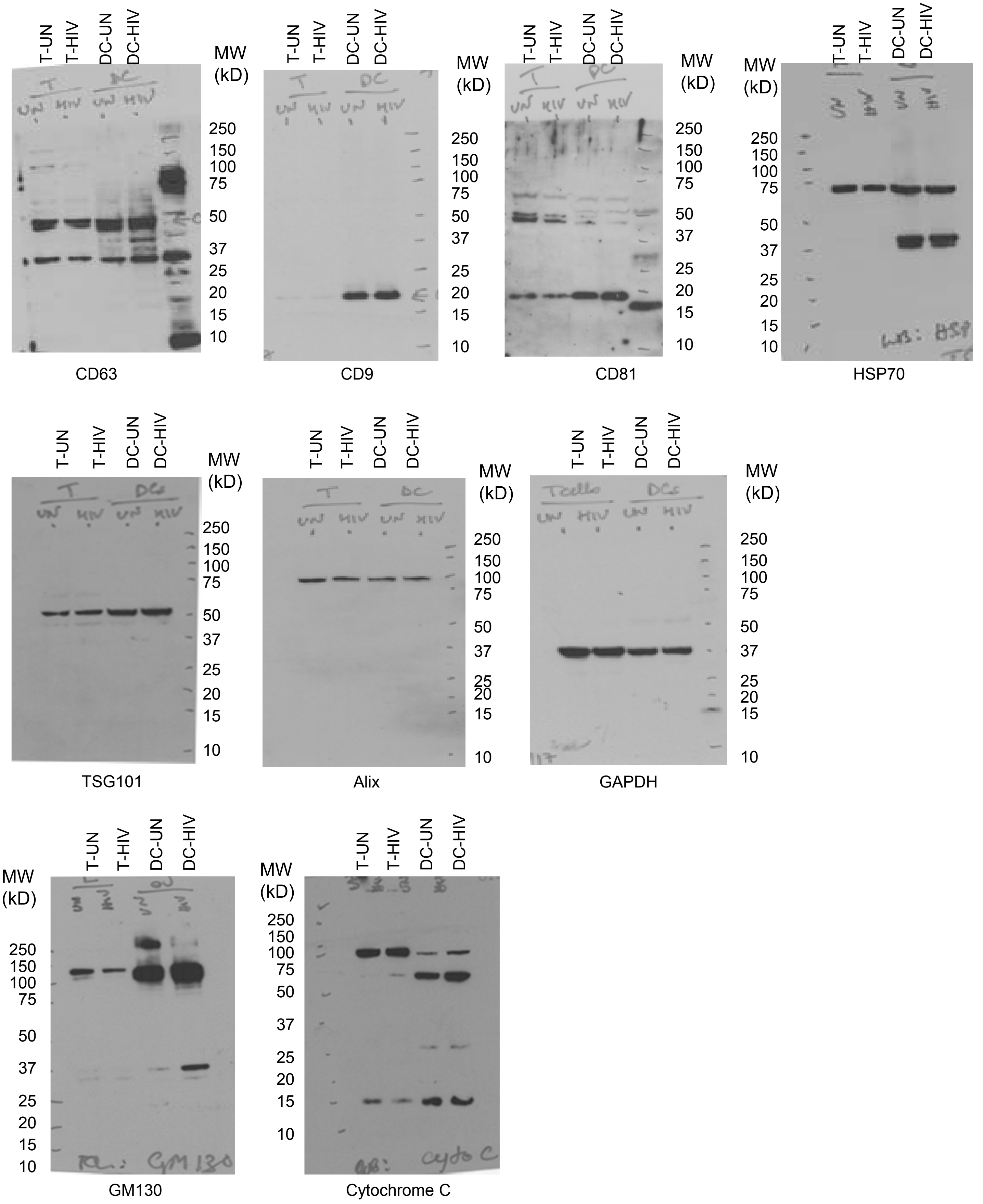

Supplementary Fig. S3C: Full-length blots for Figure 3A.

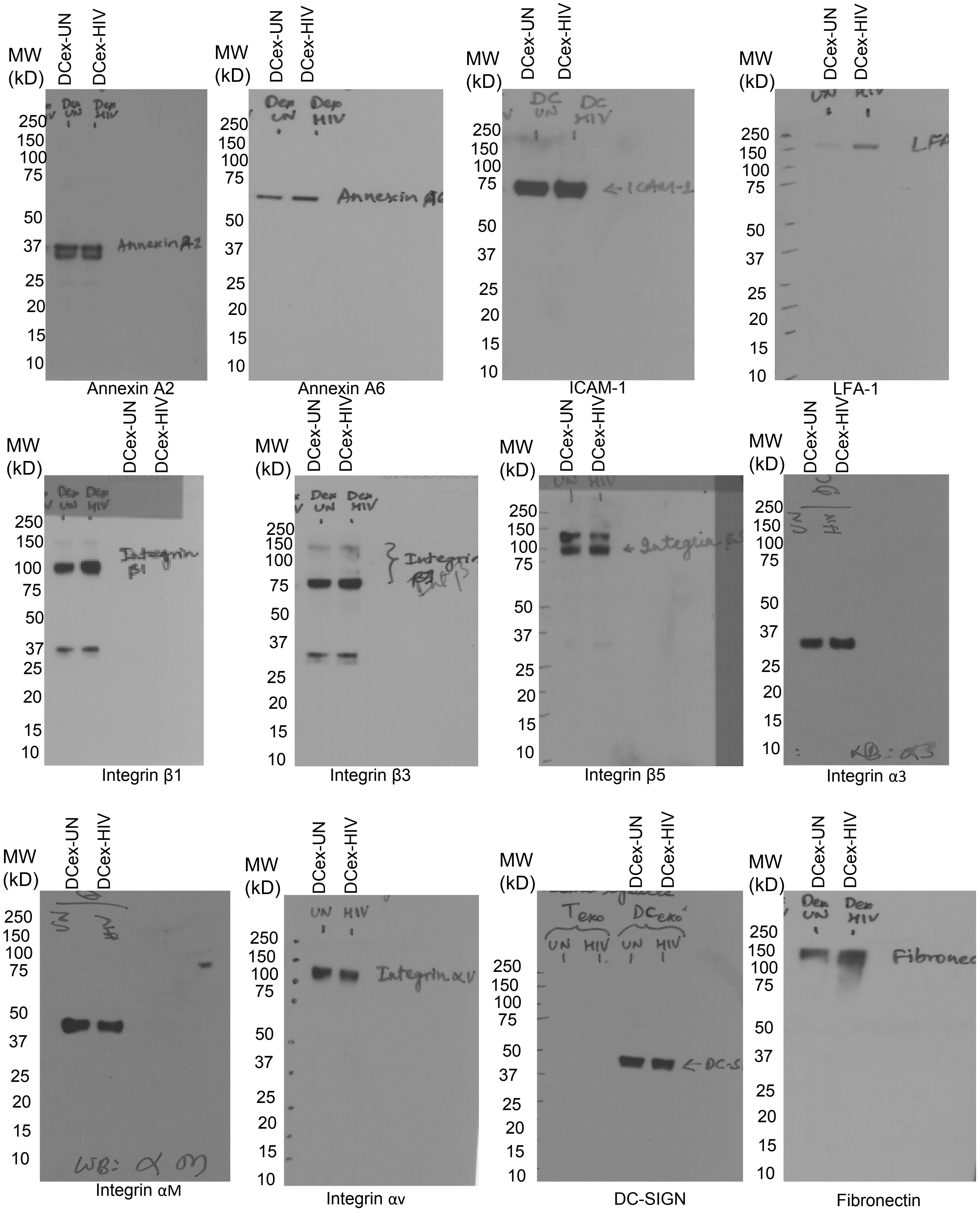

Supplementary Fig. S3C: Full-length blots for Figure 3A and C.

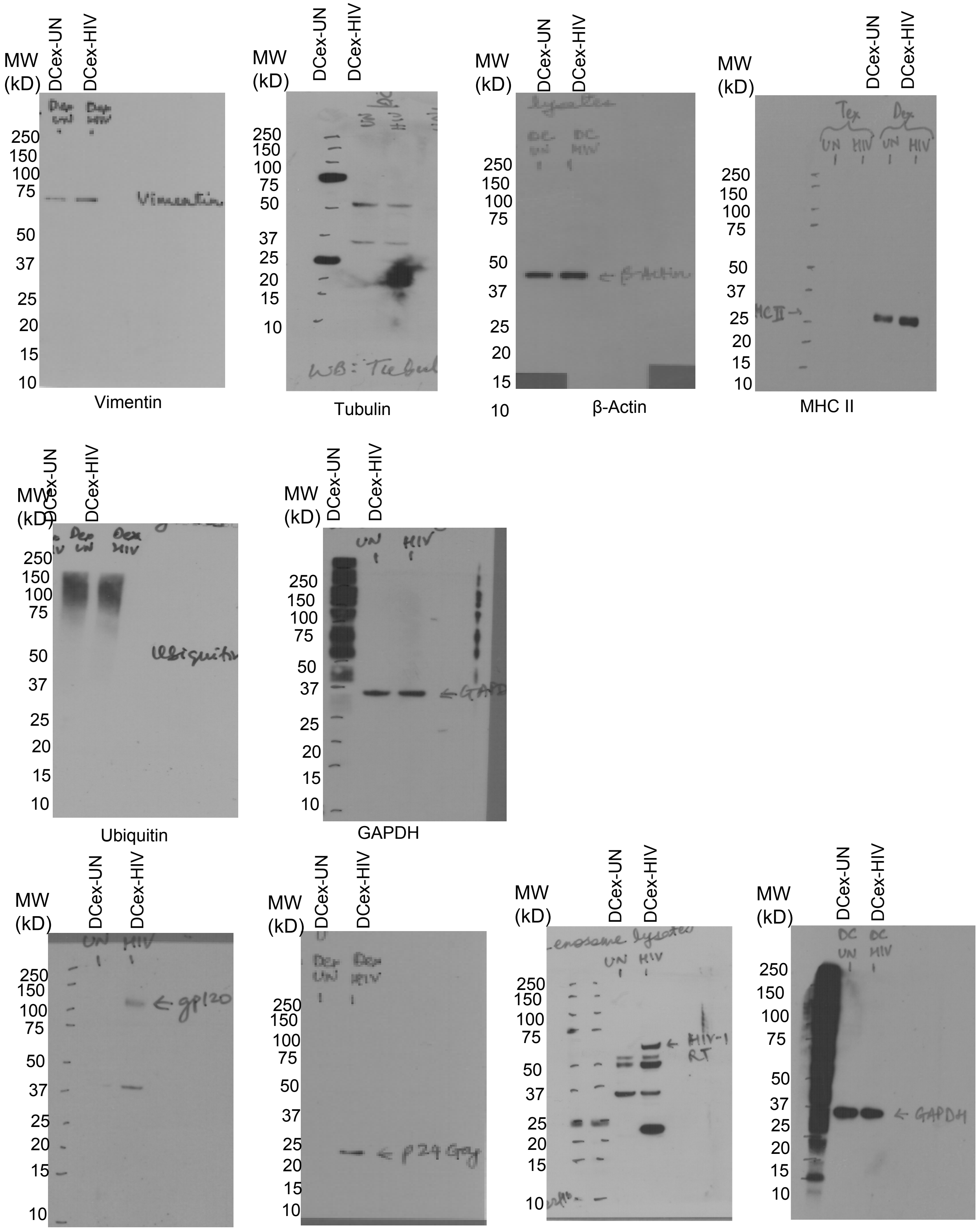

Supplementary Fig. S3D: Full-length blots for Supplemenatry Figure S1.

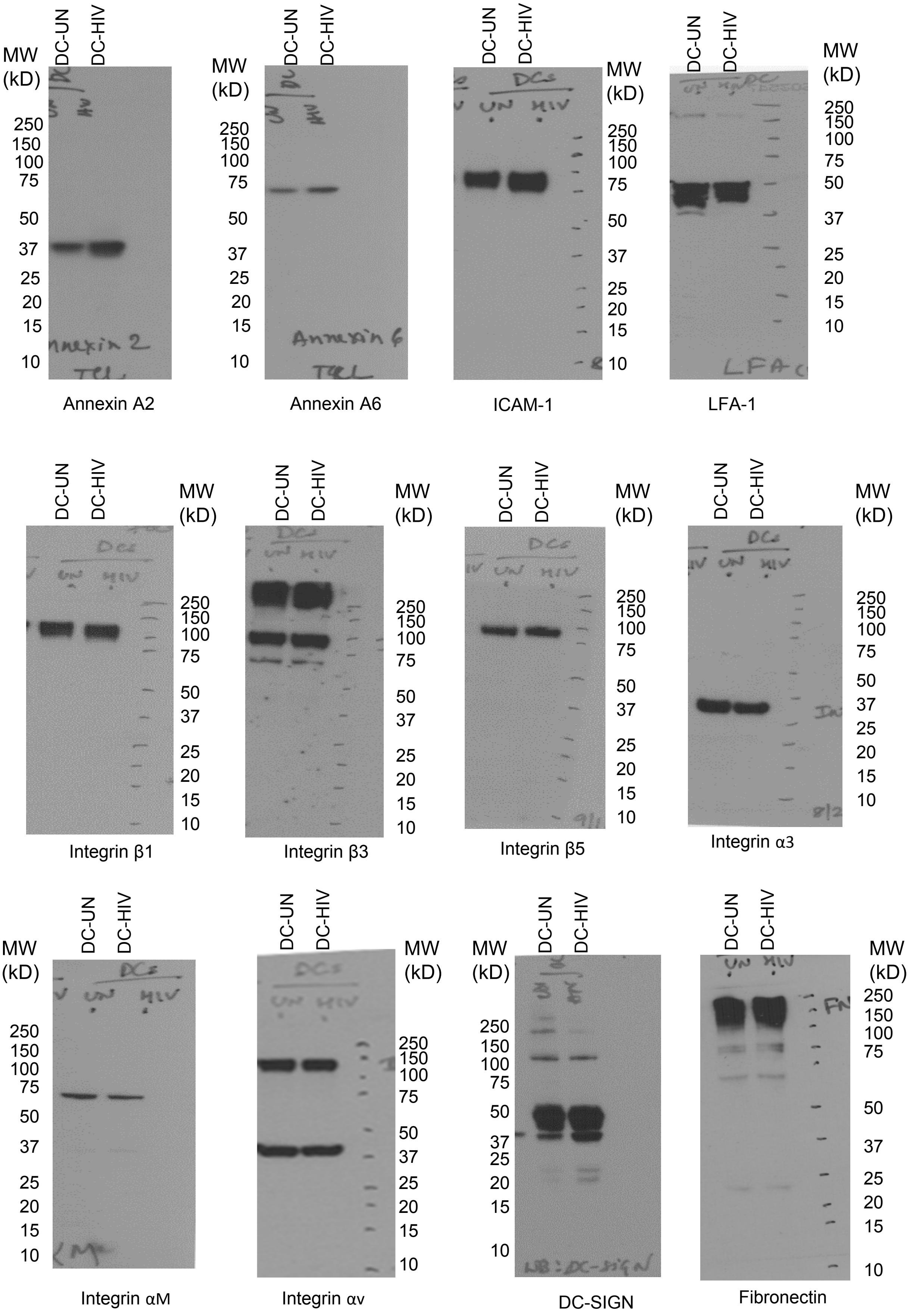

Supplementary Fig. S3D: Full-length blots for Supplemenatry Figure S1.

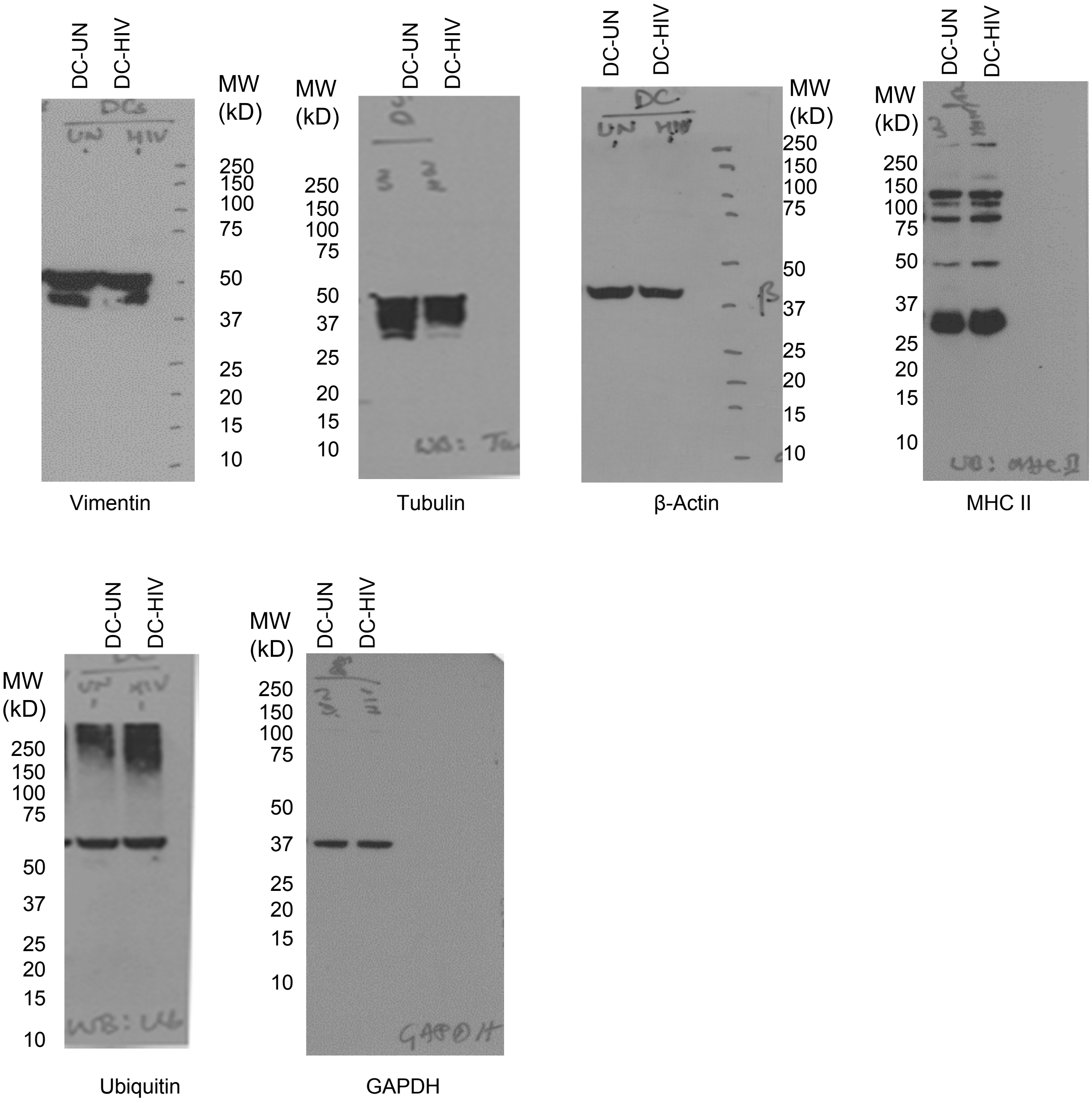

Supplementary Fig. S3E: Full-length blots for Figure 4A.

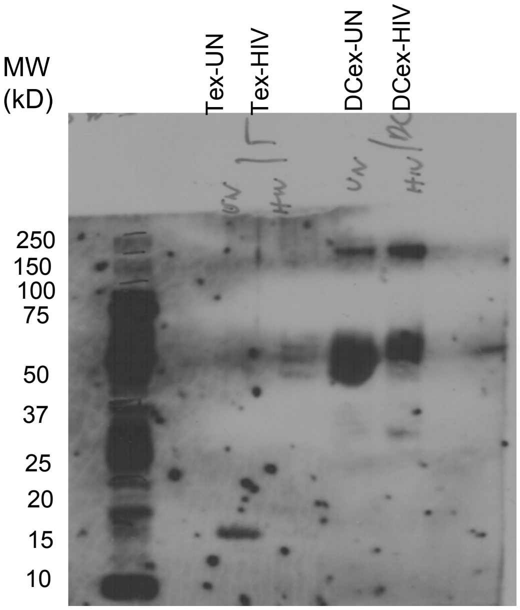

Fibronectin

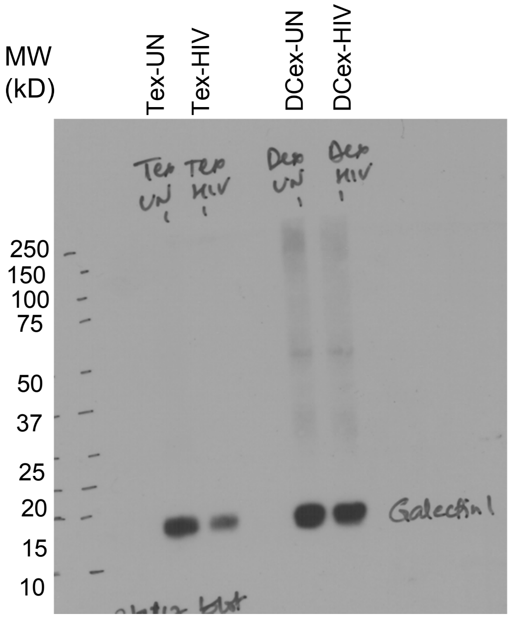

Galectin-1

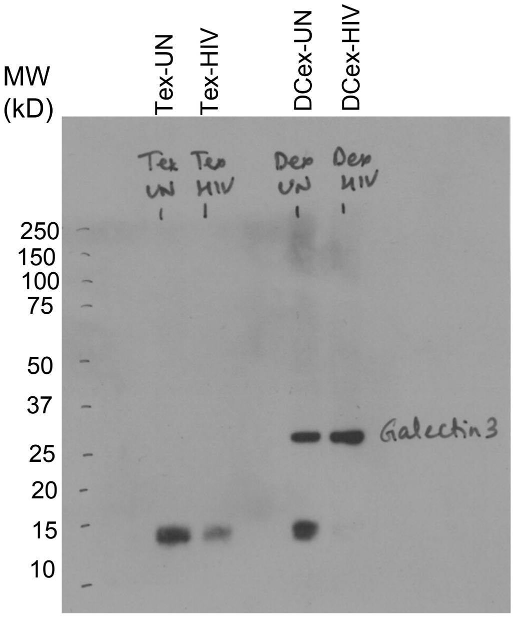

Galectin-3

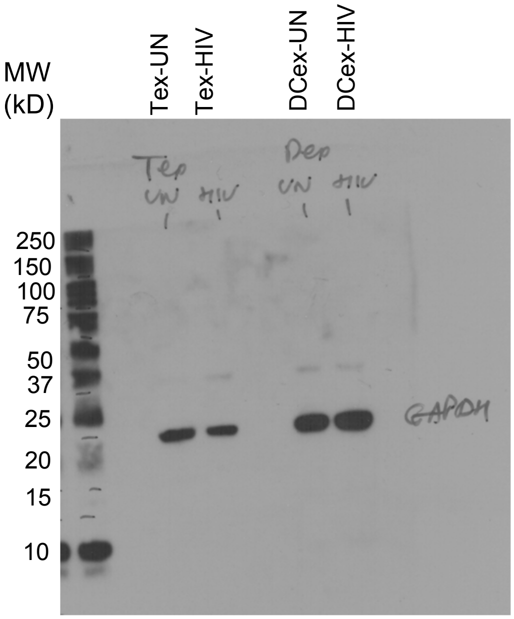

GAPDH

Supplementary Fig. S3F: Full-length blots for Figure 4C.

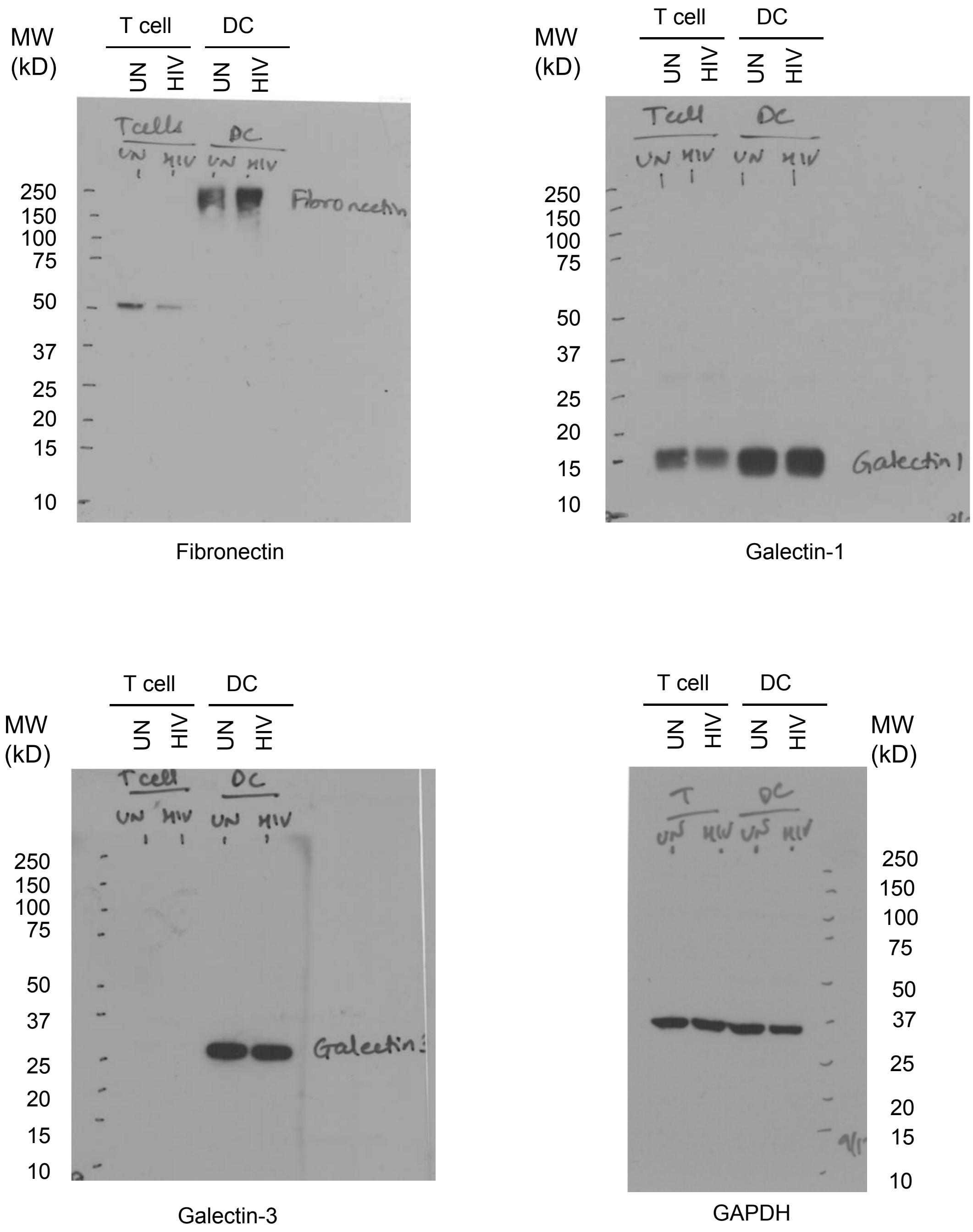

Supplementary Fig. S3G: Full-length blots for Figure 5A.

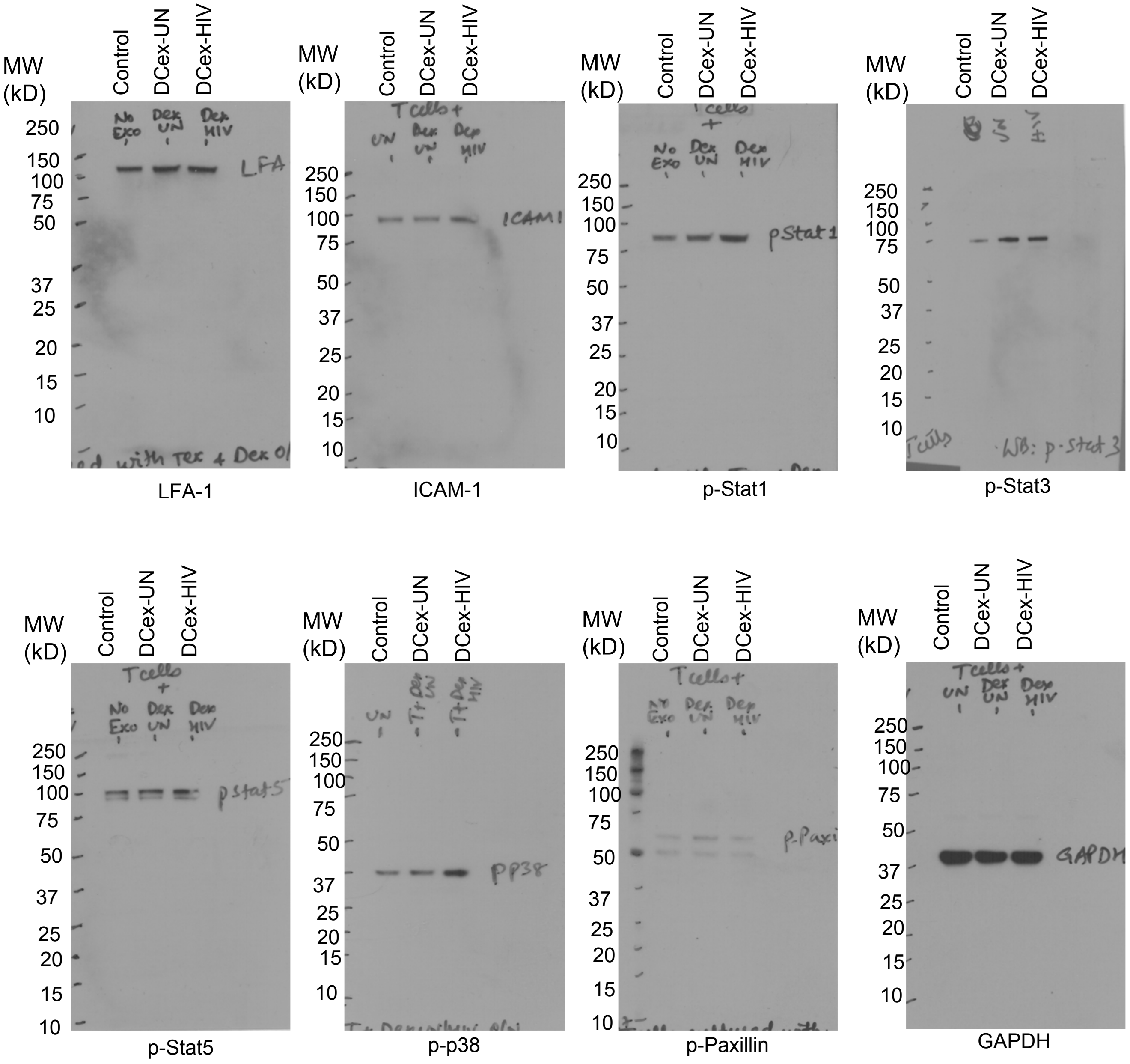

Supplementary Fig. S3H: Full-length blots for Figure 5C.

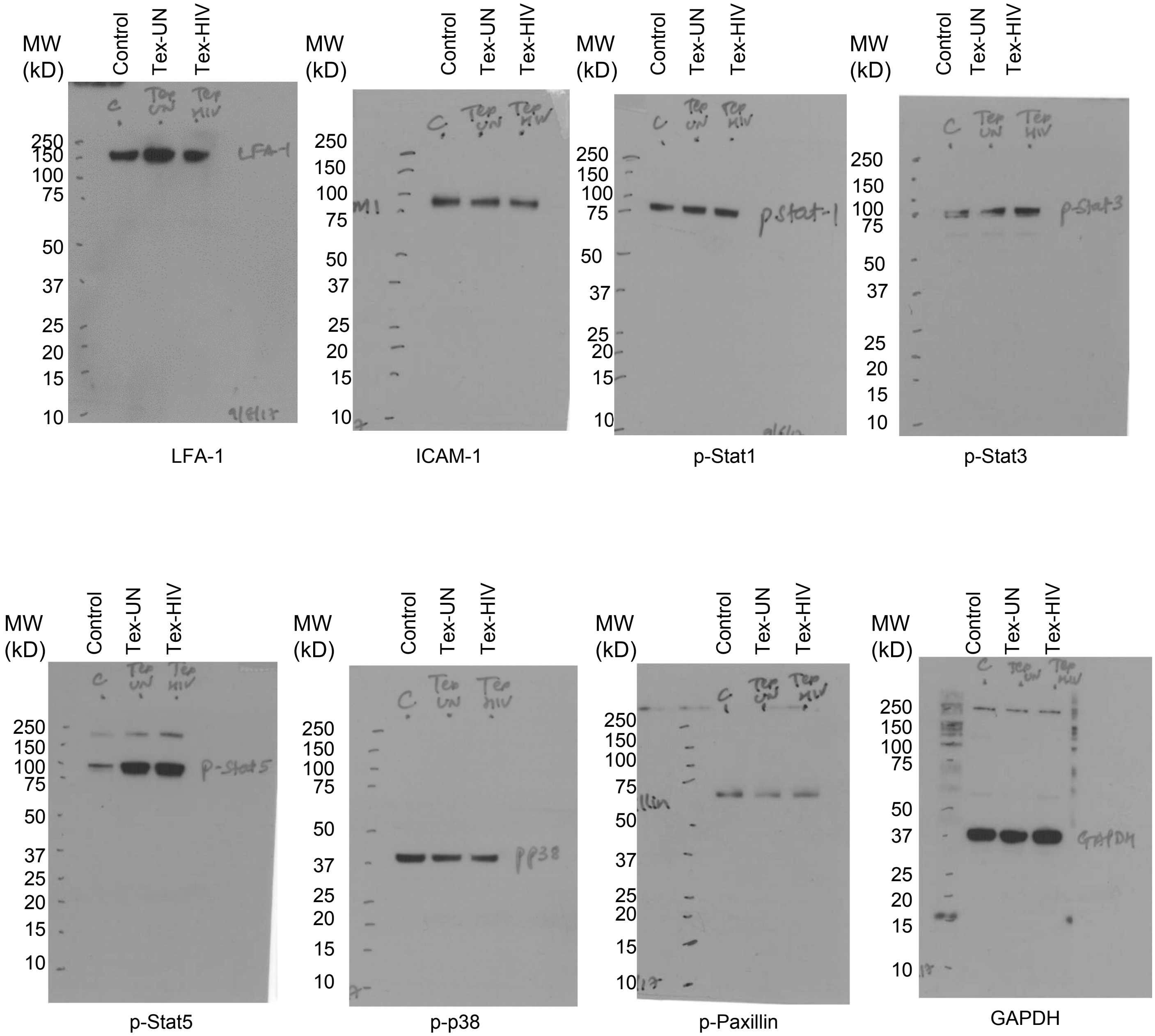

Supplementary Fig. S3I: Full-length blots for Figure 6A.

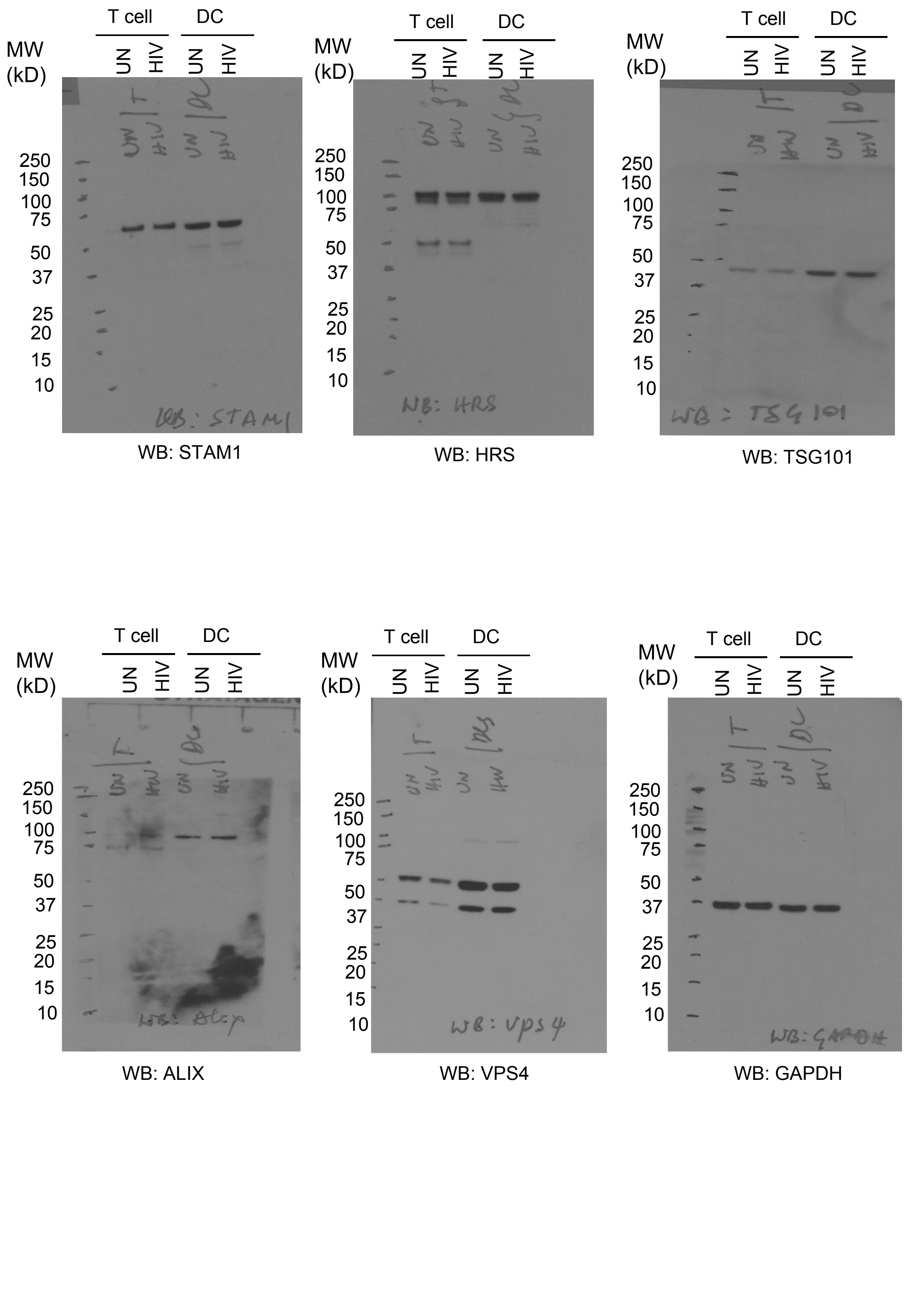

Supplementary Fig. S3I: Full-length blots for Figure 6D.

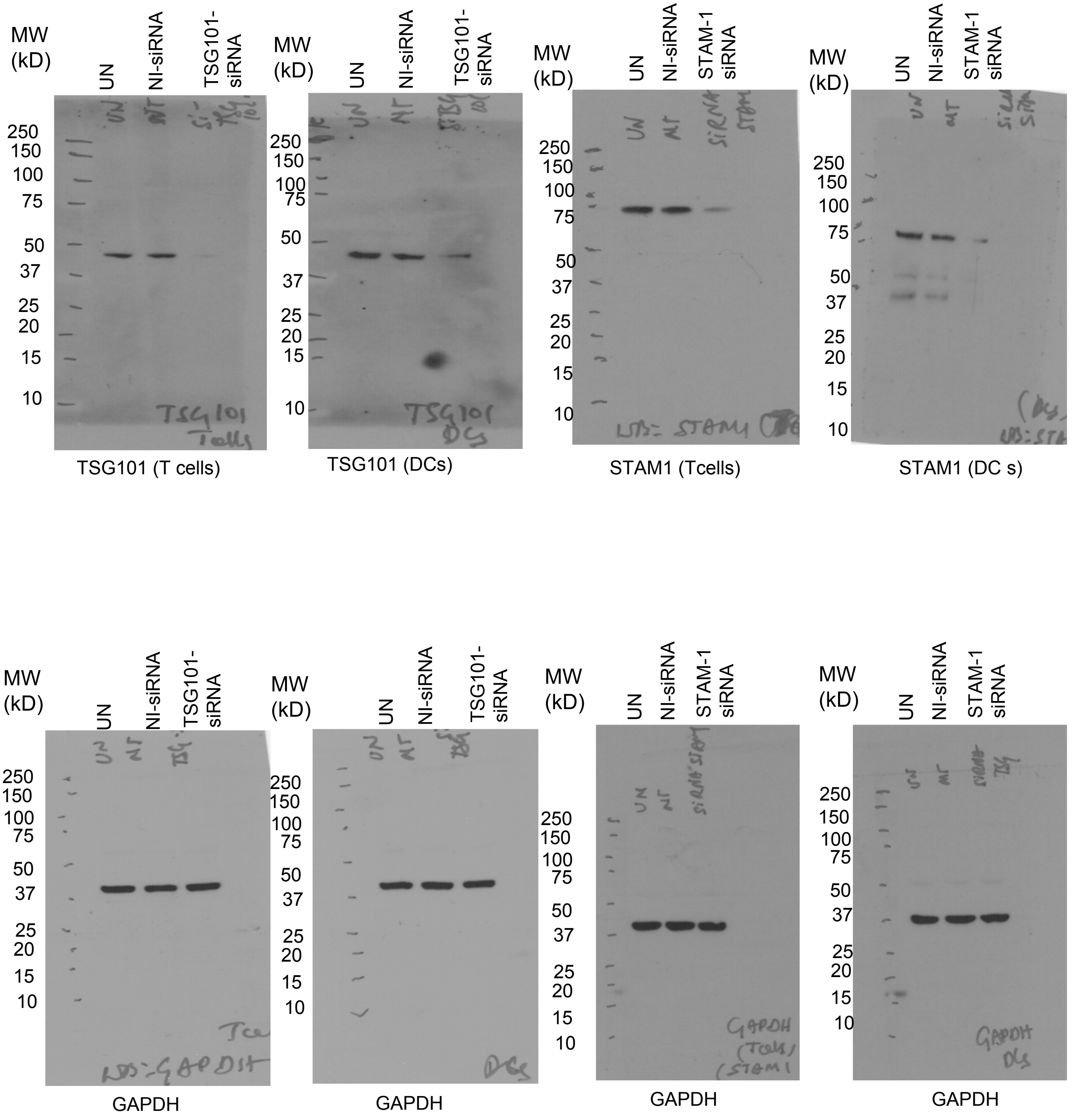

Supplement: Supplementary file 1 — Supplementary Figures [file 41598_2017_14817_MOESM1_ESM.pdf]
